# On the feasibility of all-solid-state batteries with LLZO as a single electrolyte

**DOI:** 10.1038/s41598-022-05141-x

**Published:** 2022-01-21

**Authors:** Kostiantyn V. Kravchyk, Dogan Tarik Karabay, Maksym V. Kovalenko

**Affiliations:** 1 Laboratory for Thin Films and Photovoltaics, Empa-Swiss Federal Laboratories for Materials Science and Technology, Überlandstrasse 129, 8600 Dübendorf, Switzerland; 2 Laboratory of Inorganic Chemistry, Department of Chemistry and Applied Biosciences, ETH Zürich, Vladimir-Prelog-Weg 1, 8093 Zurich, Switzerland

**Keywords:** Energy storage, Batteries

## Abstract

Replacement of Li-ion liquid-state electrolytes by solid-state counterparts in a Li-ion battery (LIB) is a major research objective as well as an urgent priority for the industry, as it enables the use of a Li metal anode and provides new opportunities to realize safe, non-flammable, and temperature-resilient batteries. Among the plethora of solid-state electrolytes (SSEs) investigated, garnet-type Li-ion electrolytes based on cubic Li_7_La_3_Zr_2_O_12_ (LLZO) are considered the most appealing candidates for the development of future solid-state batteries because of their low electronic conductivity of *ca*. 10^−8^ S cm^−1^ (RT) and a wide electrochemical operation window of 0–6 V vs. Li^+^/Li. However, high LLZO density (5.1 g cm^−3^) and its lower level of Li-ion conductivity (up to 1 mS cm^−1^ at RT) compared to liquid electrolytes (1.28 g cm^−3^; ca. 10 mS cm^−1^ at RT) still raise the question as to the feasibility of using solely LLZO as an electrolyte for achieving competitive energy and power densities. In this work, we analyzed the energy densities of Li-garnet all-solid-state batteries based solely on LLZO SSE by modeling their Ragone plots using LiCoO_2_ as the model cathode material. This assessment allowed us to identify values of the LLZO thickness, cathode areal capacity, and LLZO content in the solid-state cathode required to match the energy density of conventional lithium-ion batteries (ca. 180 Wh kg^−1^ and 497 Wh L^−1^) at the power densities of 200 W kg^−1^ and 600 W L^−1^, corresponding to *ca*. 1 h of battery discharge time (1C). We then discuss key challenges in the practical deployment of LLZO SSE in the fabrication of Li-garnet all-solid-state batteries.

## Introduction

In a search for non-flammable and non-toxic energy storage systems that possess high energy and power densities, all-solid-state batteries based on Li_7_La_3_Zr_2_O_12_ (LLZO) solid-state electrolyte (SSE) have come into a major research spotlight^1–13^. LLZO SSE has numerous advantages such as low electronic conductivity of *ca*. 10^–8^ S cm^–1^ (RT)^14^, high chemical and thermal stabilities. Moreover, contrary to other Li-ion conducting SSEs, LLZO has a wide electrochemical voltage window of 0–6 V vs. Li^+^/Li (obtained in the experimental operation)^15^, enabling its employment in combination with the metallic lithium anode and utilization of high-voltage cathodes.

**Figure 1:**
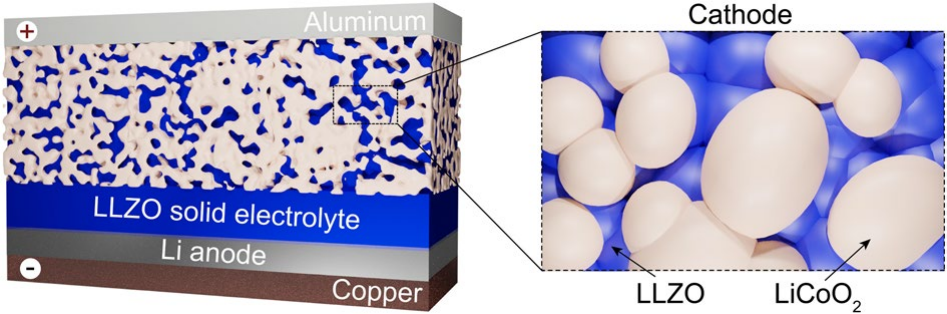
Schematics of Li-garnet solid-state battery and solid-state cathode considered in this work for assessing the power and energy densities of Li-garnet SSBs.

While broad attention to Li-garnet solid-state batteries (SSBs) is surely helpful for the progress in this area^16–20^, analysis of the literature appoints to the lack of clear opinion in the research community as to the configuration of future Li-garnet SSBs. On the one hand, the researchers are mostly united with the idea that LLZO SSE should face Li metal anode, enabling to mitigate the formation of the Li dendrites (when necessary pressure is applied)^21–25^. On the other hand, opinions on the design of the cathode layer are more divergent. Proposed are additions of LLZO^26–34^ or other electrolytes (ionic liquids^35–39^, or polymers^40^). Considering the ongoing debates on compelling Li-garnet SSB configurations, we first analyze the very first battery structure containing LLZO as the only SSE, as schematically depicted in Fig. 1. Specifically, we intend to shed light on whether this approach is feasible, given high LLZO density (5.1 g cm^−3^) and a mediocre level of its Li-ion conductivity as compared to liquid electrolytes (up to 1 mS cm^−1^ at RT)^1,2,8,41^. The resulting impact of both factors requires the employment of high LLZO content in the cathode for reaching high power density, which in turn leads to the need for much higher areal capacities of solid-state cathodes as compared to the conventional counterparts for achieving same energy densities^42^. These energy/power density tradeoff considerations point to severe limitations on the LLZO content used in the solid-state cathode and cast doubt on even the feasibility of a solely LLZO approach.

In this work, taking 18650 Panasonic (231 Wh kg^−1^, 636 Wh L^−1^)^43^ and Samsung (180 Wh kg^−1^, 497 Wh L^−1^)^44^ batteries as reference systems for defining acceptable energy density/power density balance, we assess areal capacities of Li-garnet solid-state cathodes, LLZO cathode content and LLZO thicknesses required to match their energy densities at a power density of 200 W kg^−1^ and 600 W L^−1^ (corresponding to ca. 1 h of discharge) through the simulation of Ragone plots. We discuss the critical interplay between all variables along with the calculation of their respective minimal and maximal values (break-even points). Among various possible cathode materials, the choice of cathode active material was limited to LiCoO_2_, due to its high electronic and Li-ion conductivity in both lithiated and delithiated states^45–48^. We then review in detail other factors hindering the commercial deployment of Li-garnet SSBs, such as volume changes of Li upon its plaiting/stripping, and the difficulties associated with the fabrication of LLZO based solid-state cathodes, giving our perspective on these problems.

## Results and discussion

### Energy and power densities of Li-garnet SSBs

To assess achievable energy and power density of Li-garnet SSBs, 1D-isothermal lithium-ion battery model with single-ion conducting solid electrolyte developed by COMSOL Multiphysics^49,50^ was applied to simulate their electrochemical performance during discharge. All equations governing the simulation can be found in the “Methods” section. The schematic of the model is depicted in Supplementary Fig. S1. Parameters used for simulation are summarized in Supplementary Table S1. The following compositions of LCO–LLZO solid-state cathodes were modeled: 70 vol.% of LCO—30 vol.% of LLZO, 60 vol.% of LCO—40 vol.% of LLZO, 50 vol.% of LCO—50 vol.% of LLZO and 40 vol.% of LCO—60 vol.% of LLZO. Of note, in these simulations, we considered that no interfacial resistance is present at both LLZO/Li and LLZO/LCO interfaces, and no porosity was assumed to exist in the solid-state cathodes. Clearly, these conditions are oversimplified, but they enable to assess the highest reachable limits of energy and the power density of Li/LLZO/LCO SSBs. The simulation data obtained for the exemplary systems considering LLZO/Li (0.1 Ω cm^2^)^51^ and LLZO/LCO (50 Ω cm^2^)^52^ interfacial resistances, as well as the porosity in LCO/LLZO solid-state cathode, can be found in the Supporting Information.

**Figures 2:**
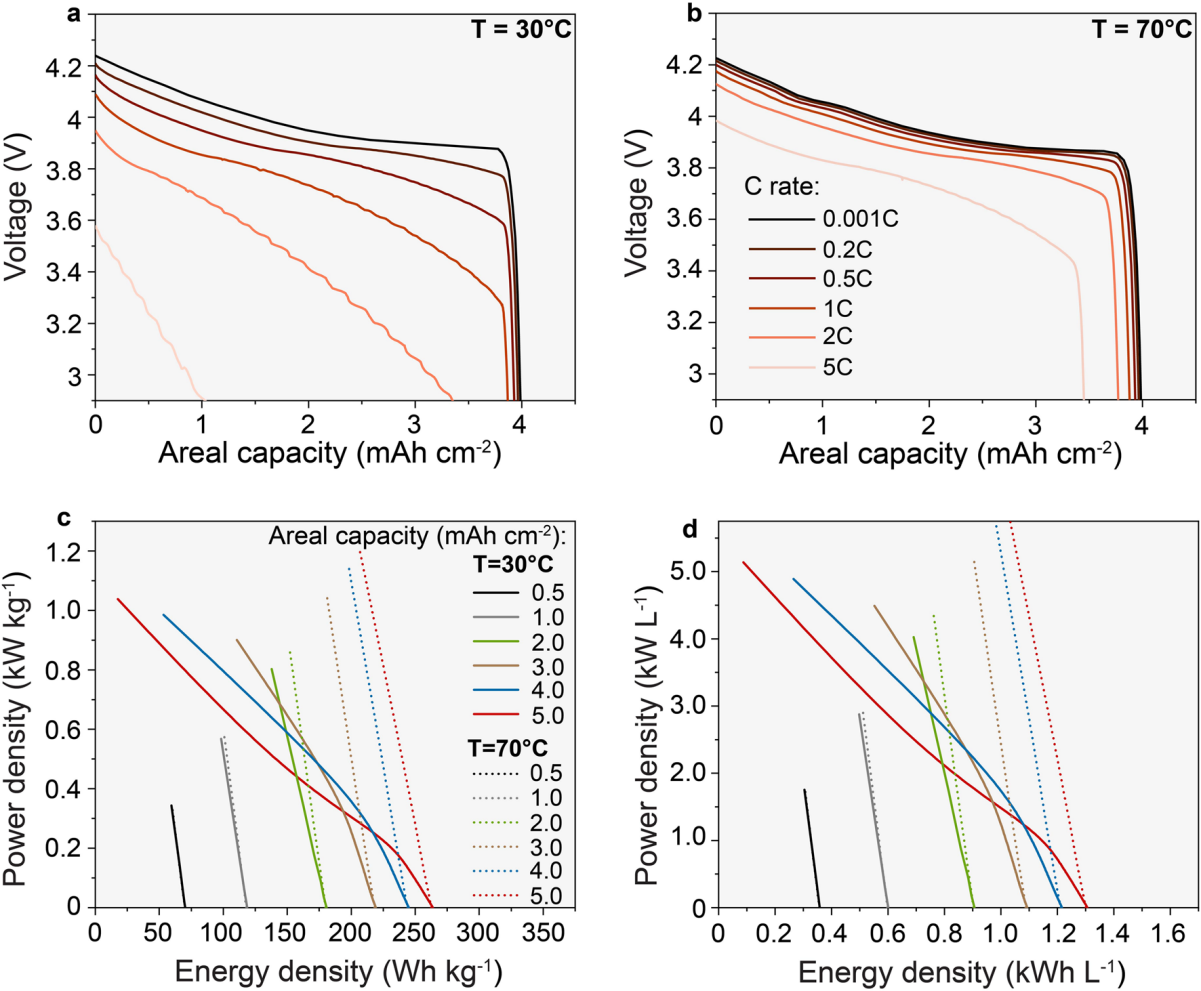
(**a**,**b**) Simulated voltage profiles of Li/LLZO/LCO all-solid-state battery at different C rates (0.001C, 0.2C, 0.5C, 1C, 2C, and 5C) and temperatures (30 °C and 70 °C), using cathode constant areal capacity of 4 mAh cm^−2^ and LLZO thickness of 30 µm. (**c**,**d**) Gravimetric and volumetric Ragone plots of Li/LLZO/LCO solid-state battery simulated using cathode areal capacities of 0.5, 1, 2, 3, 4, and 5 mAh cm^−2^ at temperatures of 30 °C and 70 °C and constant LLZO thickness of 30 µm. The simulations were performed using LCO cathode that is composed of 70 vol.% of LCO and 30 vol.% of LLZO.

Simulations were performed varying (i) the areal capacity of the cathode layer (0.5–5 mAh cm^−2^), (ii) the thickness of LLZO dense layer separating the cathode and Li anode layers (0.1–90 µm), (iii) the temperature (30 °C and 70 °C), and (iv) the C-rate (0.5C–5C). Examples of the resulting voltage profiles simulated at different C-rates, cathode areal capacity of 4 mAh cm^−2^, and LLZO thicknesses of 30 µm are shown in Fig. 2a,b (see Supplementary Figs. S2–S25, for a complete set of simulated voltage profiles). The achievable energy and power densities were calculated from simulated voltage profiles at a specific C-rate and cathode mass loadings (areal capacities), average cell voltages, and total weight or volume of all cell components assuming a combination of 40 cathode/electrolyte/anode layers (see Supplementary Fig. S26 for details). Of note, the cell volume is calculated in the fully discharged state in which Li-garnet SSBs are to be assembled in practice. All parameters contributing to the mass and volume of the cell, such as the thickness of Cu and Al foils and the thickness of pouch Al foil are summarized in Supplementary Table S2. Thickness of the Li metal anode was fixed, corresponding to 20% of the cathode areal capacity. For instance, Li anode thickness was 1, 3, and 5 µm for cathodic areal capacities of 1, 3, and 5 mAh cm^−2^, accordingly. It should also be pointed out that efficient operation of Li-garnet SSBs with high areal capacities of > 1 mAh cm^−2^ can be practically achieved only at stack pressures that enable to mitigate the formation of voids at the LLZO/Li interface^21–24^. As a result, the cell design requires integrating additional inactive components, which severely limits its energy density. Considering the lack of data on the optimal pressure at given current densities and the practical implementation of this requirement, we excluded this parameter from the energy and power density calculations.

Figure 2c,d show gravimetric and volumetric Ragone plots of Li-garnet SSBs composed of LCO cathode with 30 vol.% of LLZO at different cathode mass loadings corresponding to the theoretical areal capacities of 0.5–5 mAh cm^−2^ (at 30 °C and 70 °C) and at fixed thickness of LLZO solid electrolyte of 30 µm. Ragone plots for other thicknesses of 0.1, 10, 60, and 90 µm are shown on Supplementary Figs. S27–S28.

**Figure 3:**
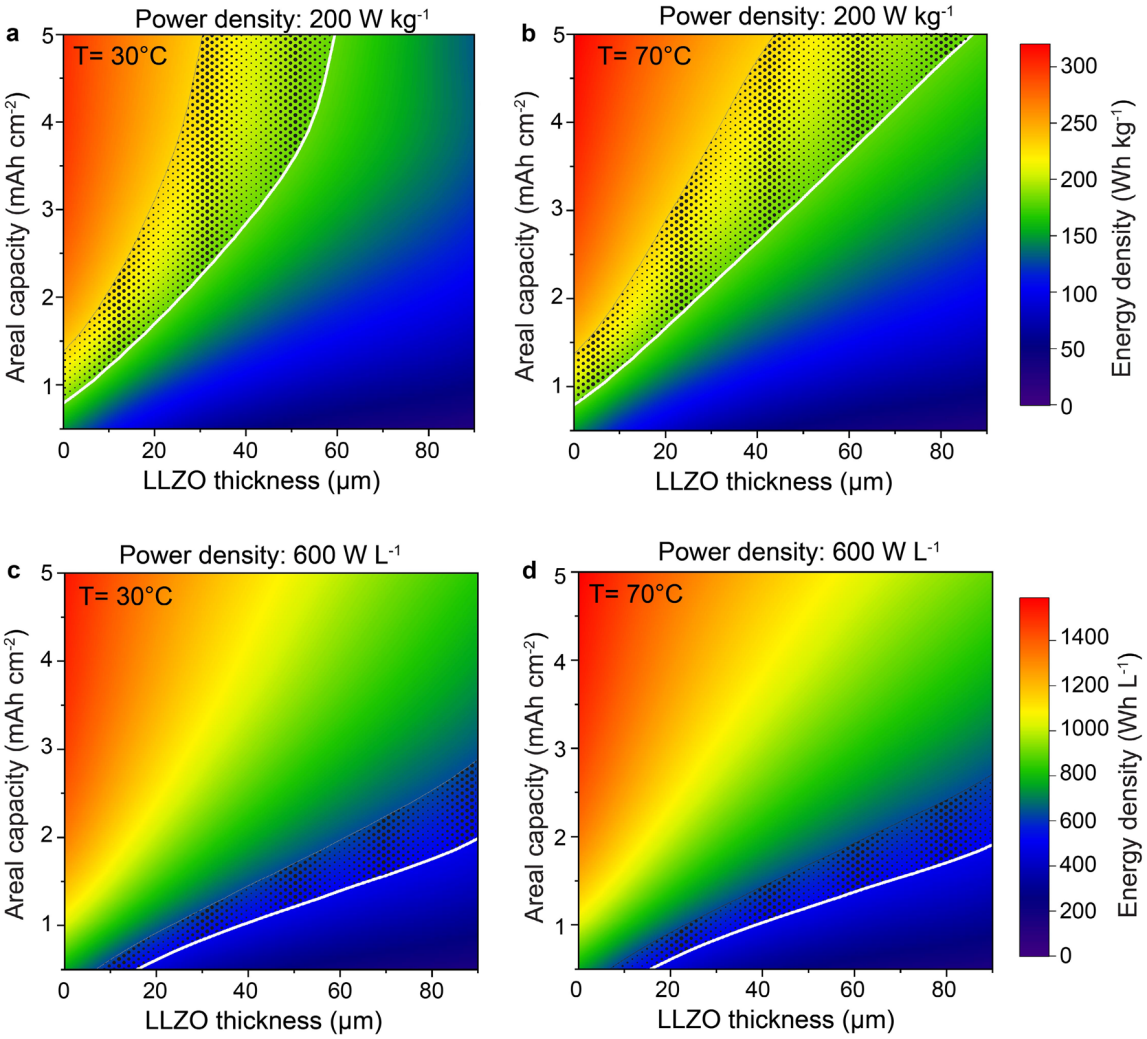
Simulated gravimetric (**a**,**b**) and volumetric (**c**,**d**) energy densities of Li/LLZO/LCO all-solid-state battery vs. cathode areal capacity and LLZO thickness plotted at power densities of 200 W kg^−1^ and 600 W L^−1^ and temperatures of 30 °C and 70 °C. The composition of LCO cathode is constant (70 vol.% of LCO and 30 vol.% of LLZO). The areal capacity (thickness) of the Li metal anode corresponds to 20% of the cathode areal capacity. The gravimetric and volumetric energy densities of Li-free/LLZO/LCO all-solid-state battery (so-called Li-free configuration), are shown in Supplementary Fig. S29 for comparison.

Next, we analyzed changes of the energy density of the studied solid-state system at gravimetric and volumetric power densities of 200 W kg^−1^ and 600 W L^−1^, corresponding to *ca*. 1 h of a full discharge. The data derived from Fig. 2c,d and Supplementary Figs. S27–S28 were plotted in the form of 3D maps governing the relationship between areal capacities of LCO cathodes, LLZO thicknesses, and the energy density (Fig. 3). Additionally, state-of-the-art values for conventional LIBs are shown in Fig. 3 as mesh area, where upper and lowest levels correspond to the energy densities of 18650 Panasonic (231 Wh kg^−1^, 636 Wh L^−1^)^43^ and Samsung (180 Wh kg^−1^, 497 Wh L^−1^)^44^ LIBs at the gravimetric and volumetric power densities of 200 W kg^−1^ and 600 W L^−1^, accordingly. Considering 18650 Samsung battery as a reference system with the minimum necessary electrochemical performance, we identify LLZO thicknesses and areal capacities of Li-garnet SSBs required to match its energy density at 200 W kg^−1^ and 600 W L^−1^. Such values were named break-even thickness and areal capacities, following analogy to the break-even values in economics, representing a set of parameters at which total revenue and total expenses are equal. The break-even areal capacity-thicknesses curves for 30 °C and 70 °C are indicated in white. As follows from Fig. 3a, at 30 °C, gravimetric break-even LLZO thickness ranges from 5.5 to 59.5 µm for 1 mAh cm^−2^ and 5 mAh cm^−2^, accordingly. However, in the case of 70 °C, higher LLZO thicknesses of 5.8 to 86.9 µm (1 mAh cm^−2^–5 mAh cm^−2^) can be used in order to reach the same energy density of 180 Wh kg^−1^ at 200 W kg^−1^. The volumetric break-even LLZO thicknesses were very similar at both temperatures: 15.8–66.2 µm and 15.7–67.5 µm for 0.5–1.5 mAh cm^−2^ at 30 and 70 °C, accordingly.

Notably, considering the employing of a 50 µm LLZO solid-electrolyte membrane, the gravimetric break-even cathode areal capacity equals 3.55, and 3.15 mAh cm^−2^ for the cells at 30 °C and 70 °C. Volumetric areal capacity of 1.2 mAh cm^−2^ was found to be the break-even value for both temperatures of 30 °C and 70 °C. Importantly, our calculations show that an increase of the Li anode excess has a moderate impact on the gravimetric energy density of Li-garnet SSBs (Supplementary Fig. S30a,b). For instance, when Li anode thickness was enlarged by a factor of 10, the gravimetric break-even LLZO thickness at a cathode areal capacity of 1.5 mAh cm^–2^ shifted only from 16.8 to 14.9 μm and from 16.8 to 15.4 μm at 30 °C and 70 °C, accordingly. Correspondingly, at a fixed cathode areal capacity of 1.5 mAh cm^−2^ and LLZO thickness of 15 μm, the gravimetric energy density decreases from 184.4 Wh kg^−1^ and 185.8 Wh kg^−1^ to only 180.2 Wh kg^−1^ and 181.2 Wh kg^−1^ (at 30 °C and 70 °C). With respect to the volumetric energy density, the picture is the opposite. Ten times higher Li anode amount changes significantly volumetric break-even thicknesses at a cathode areal capacity of 1.5 mAh cm^–2^, from 66.2 to 53.2 μm and 67.5 to 54.3 μm (at 30 °C and 70 °C, see Supplementary Fig. S30c,d). As a result, a substantial reduction of the volumetric energy density was found in the case of employing a 10-times thicker Li anode (at a fixed cathode areal capacity of 1.5 mAh cm^−2^ and LLZO thickness of 15 μm): from 939 Wh L^−1^ to 774 Wh L^−1^ for 30 °C and from 942 Wh L^−1^ to 777 Wh L^−1^ for 70 °C.

**Figure 4:**
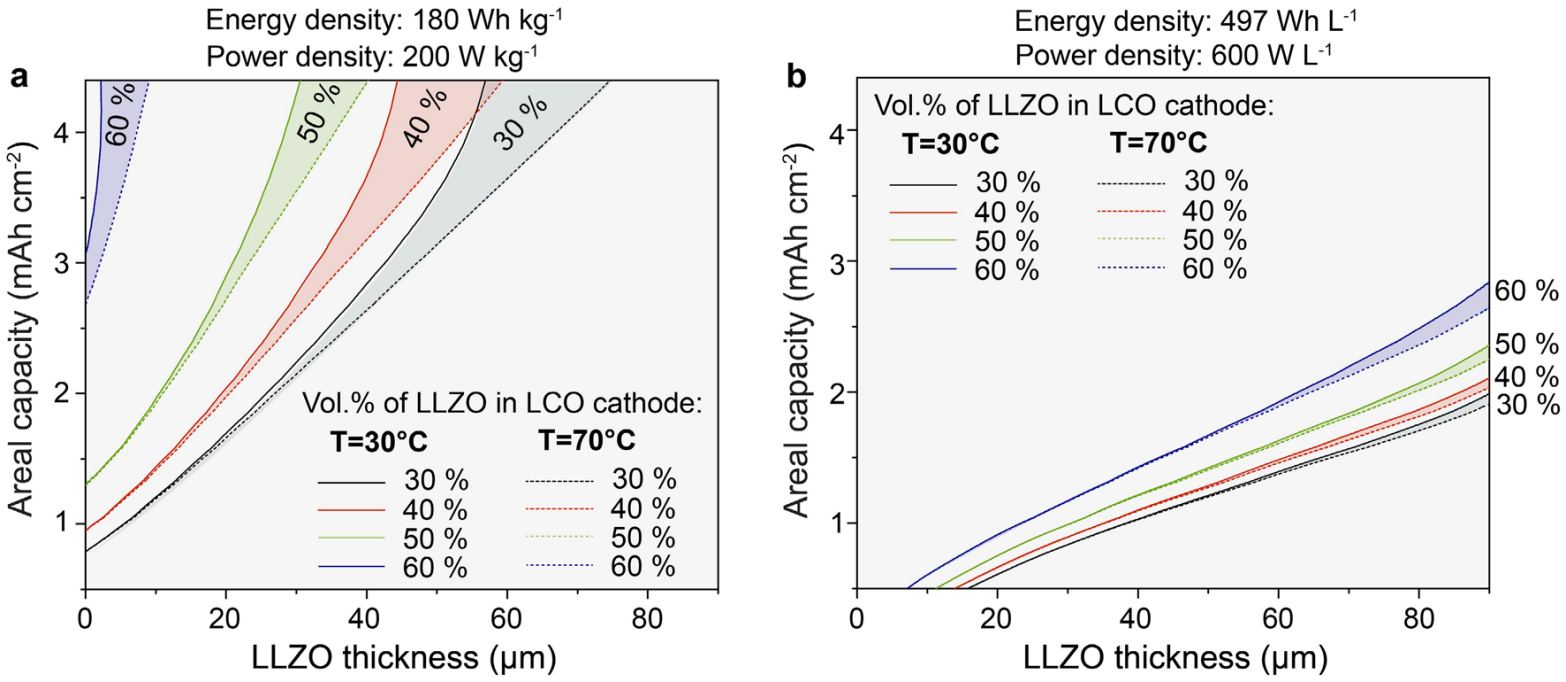
Gravimetric and volumetric break-even dependence of cathode areal capacity and LLZO thickness for Li/LLZO/LCO solid-state battery comprising LCO cathode with different volumetric content of LLZO. These dependencies were derived from simulated voltage profiles shown on Supplementary Figs. S2–S7, S8–S13, S14–S19, and S20–S25 and corresponding Ragone plots shown on Supplementary Figs. S27–S28, S31–S32, S33–S34, and S35–S36 (for 30, 40, 50, and 60 vol. % of LLZO in LCO). Complete sets of data of **s**imulated energy densities are shown on Fig. 3 (70 vol.% of LCO and 30 vol.% of LLZO), Supplementary Fig. S37 (60 vol.% of LCO and 40 vol.% of LLZO), Supplementary Fig. S38 (50 vol.% of LCO and 50 vol.% of LLZO), and Supplementary Fig. S39 (40 vol.% of LCO and 60 vol.% of LLZO).

Subsequently, we analyzed break-even dependences of cathode areal capacity and LLZO thickness for Li/LLZO/LCO solid-state battery on the vol. % of LLZO in LCO solid-state cathode, which are summarized in Fig. 4. Figure 4a evidences that upon an increase of LLZO content in a composite cathode, higher areal capacity and lower LLZO solid electrolyte thickness should be used to attain the same energy densities of 180 Wh kg^−1^ and 497 Wh L^−1^ at gravimetric and volumetric power densities of 200 W kg^−1^ and 600 W L^−1^. Interestingly, assuming that the Li-garnet SSBs can be fabricated with 50 µm thick LLZO membrane, 50 vol.% of LLZO fraction in LCO cathode results in gravimetric energy densities not matching the energy density of a conventional Li-ion battery even at very high LCO areal capacity of 4 mAh cm^−2^, requiring LLZO thickness of < 40 µm. In the case of 60 vol.%, the maximal allowed LLZO thickness at LCO areal capacity of 4 mAh cm^−2^ equals 2 μm at 30 °C, which can be slightly increased to 7 μm at a higher temperature of 70 °C.

With regard to the volumetric performance, already at high LLZO loading of 60 vol. %, relatively thick LLZO membranes of 50–90 µm at LCO areal capacities of 1.6–2.8 mAh cm^−2^ can be employed to reach volumetric energy density of 497 Wh L^−1^ at 600 Wh L^−1^ (30 °C). Based on obtained results, below, we summarize recommended areal capacities of LLZO–LCO cathode at LLZO thickness of 10, 20, 30, and 50 µm and LLZO volumetric content of 30, 40, 50, 60% and temperatures of 30 °C or 70 °C (Table 1).

**Table 1:** Recommended areal capacities of LLZO–LCO cathode at given LLZO thickness and vol. % of LLZO in LCO solid-state cathode. Sign "–" means that at given temperature, LLZO thickness and LLZO content in the cathode, the Li-garnet SSB possess lower energy density values than the state-of-the-art value of 180 Wh kg^−1^ at a power density of 200 W kg^−1^.

T, °C	Vol.% of LLZO in LCO cathode	Thickness of LLZO, µm	Gravimetric break-even areal capacity, mAh cm^−2^	Volumetric break-even areal capacity, mAh cm^−2^
30	30	20	1.70	0.61
		40	2.84	1.03
70	30	20	1.66	0.61
		40	2.64	1.03
30	40	20	2.04	0.67
		40	3.67	1.1
70	40	20	1.98	0.67
		40	3.18	1.1
30	50	20	2.9	0.76
		40	–	1.22
70	50	20	2.72	0.76
		40	4.39	1.21
30	60	20	–	0.91
		40	–	1.43
70	60	20	–	0.91
		40	–	1.42

### Practical challenges towards the fabrication of Li-garnet all-solid-state batteries

Apart from the importance of the above-discussed findings, which highlight the viability of solely garnet SSBs in respect of achievable energy and power densities, adopting the LLZO into the battery cell structure is challenging and requires new approaches in the fabrication of solid-state cathodes and cell design. The latter are primarily related to (i) the intrinsic volume changes of Li anode upon Li plating and stripping and (ii) the chemical reaction of cathode materials with LLZO, yielding non-Li ion conductance phases. These two factors were fully excluded from the simulations.

The volume change of Li is becoming a major challenge when it comes to the deposition of high areal capacities of 1–5 mAh cm^−2^ that correspond to 5–25 µm of Li. This means that, on the one hand, cell design should account for the dynamic expansion of Li anode upon charge. On the other hand, upon discharge, i.e., upon stripping of Li, stack pressure should be applied to the LLZO/Li interface to prevent the formation of cavities, which may arise from the insufficient rate of Li^+^ diffusion and applied pressure to replenish the Li being dissolved into LLZO^23^. Contrarily, voids can accumulate at the LLZO/Li interface leading to increased local current density and the formation of Li dendrites upon cycling. A rough estimate for the required stack pressure range, provided by Kasemchainan et al.^53^, is ca. 10 MPa, allowing to achieve stable cycling at current densities exceeding 1 mA cm^–2^. The external stack pressure is, however, a double-edged sword. Although it seems necessary to inhibit void formation during lithium stripping, some theoretical^22,54^, and experimental reports^55^ indicated that stack pressure could lead to quicker cell failure due to increased mechanical stress. Therefore, research is ongoing on finding optimum pressure for Li stripping and the development of cell design enabling to accommodate the dynamical changes in lithium thickness. It should be noted, however, that one possible solution to avoid the Li volume change issue is to use a scaffold-type LLZO, since no external pressure is needed in this case. Thus, Li metal can be plated over the entire surface of the scaffold structure and stored in the pores. As a result, there is no dynamic change in the volume of the cell upon plaiting. Upon stripping, the voids are not forming due to the high surface area of the LLZO/Li interface. Another essential advantage of LLZO scaffolds is the possibility to increase applied current density upon Li plating/stripping up to 10 mA cm^−2^ without the formation of Li dendrites. For instance, as indicated by Wachsmann et al.^56^ the current density of 10 mA cm^−2^ for porous LLZO configuration corresponds to the current density of 0.25 mA cm^−2^ for planar configuration, considering that the porous solid-state electrolyte might have up to ~ 40 × higher surface area compared to the planar one.

As to the compatibility of LLZO with current cathode chemistries, this issue is mainly caused by the high-temperature co-sintering between LLZO and cathode active materials. For instance, upon heat-treatment of LLZO and LiCoO_2_ (LCO) cathode above 700 °C, insulating decomposition reaction products were reported^50^. High-voltage spinel cathodes (Li_2_NiMn_3_O_8_, Li_2_FeMn_3_O_8_, and LiCoMnO_4_) start to react with LLZO even at temperatures as low as 500 °C^57^. LiFePO_4_ (LFP) cathodes are difficult, if not impossible, to co-sinter with LLZO, as LFP phase decomposition already occurs above 400 °C^26^. To overcome this compatibility issue, a new design was recently proposed based on wet-chemical infiltration of the cathode active material precursors into porous LLZO solid-state electrolyte, serving as an as-sintered scaffold with their subsequent annealing at lower temperatures^26^. Alternatively, as-synthesized cathode material can be infiltrated into LLZO scaffold, as was demonstrated for LiNi_0.6_Mn_0.2_Co_0.2_O_2_ (NMC622) particles by Doeff et al.^58^ Although both methods are interesting, they result in relatively low amounts of active materials, leading to low cathode areal capacities.

## Summary and outlook

In this work, we analyzed three key contributions to the energy and power densities of Li-garnet solid-state batteries: (i) the LLZO thickness and cathode areal capacity, (ii) Li anode excess, and (iii) the content of LLZO in composite LCO cathodes. We identified the values of each variable that allow the cell to attain the energy density of conventional Li-ion batteries (180 Wh kg^−1^ and 497 Wh L^−1^) at the power density of 200 W kg^−1^ and 600 W L^−1^, corresponding to *ca*. 1 h of a full discharge. In short, our findings indicate the viability of solely garnet SSB configuration. The detailed conclusions and recommendations are as follows.

First, we reiterate that researchers should focus on Li-garnet SSBs with a small LLZO solid electrolyte thickness of 20–50 µm rather than continue reporting on mm-thick LLZO pellets. Only when employing such thin LLZO membranes the state-of-the-art gravimetric and volumetric energy densities of conventional Li-ion batteries at 1 h of battery discharge can be attained. Also, working with such thin LLZO thicknesses will aid in identifying at early stages technical challenges in their mass-scale manufacturing. We further note that at LLZO SSE thickness of > 50 µm the employment of cathodes with high areal capacities of > 3 mAh cm^−2^ is imperative for reaching high gravimetric energy densities of 180 Wh kg^−1^ at a power density of 200 Wh kg^−1^. Thus far, the laboratory LLZO-based cells utilized electrodes with very low areal capacities (on average 0.5–1 mAh cm^−2^).

Our analysis also concerns the thickness of the Li-metal anode and the content of LLZO in the composite cathodes. Briefly, our calculations show that minimization of the Li excess in Li-garnet SSBs is not very critical for its gravimetric energy density. The employment of thick commercial Li/Cu foils with the Li thickness of *ca*. 50 µm still appears to be a practically viable approach. The content of LLZO in LCO solid-state cathodes should be minimized at least to the level of 30–50 vol.% as higher LLZO content requires the deployment of very thin, 2–7 µm LLZO membranes, which is not feasible to achieve experimentally. Additionally, we present recommended minimal areal capacities of LLZO–LCO cathode at given LLZO thickness and vol. % of LLZO in LCO solid-state cathode.

Apart from the cathode loadings, LLZO content in the solid-state cathode, the thicknesses of LLZO SSE, and the metallic Li anode that needs to be adjusted, we highlight that also other aspects of the Li-garnet SSB fabrication should be considered. They are the intrinsic volume changes of Li anode upon its plating and stripping and challenges related to the fabrication of LLZO composed solid-state cathodes. Although it seems that the volume changes of the Li at the anode side can be mitigated either by the employment of pressure or the use of scaffold-type LLZO membranes, it is not yet certain whether it is possible to fabricate solid-state cathodes based on LLZO and Li transition metal oxides, considering their incompatibility upon co-sintering or difficulty in the infiltration of cathode particles in LLZO porous scaffold.

## Methods

### Simulations of LCO positive electrode

#### Electronic conductivity in LCO

The conduction of electrons in the LCO can be described by following generalized Ohm’s Law:

1 $${i}_{LCO}=-{\upsigma }_{LCO,eff}\nabla {\mathrm{\varnothing }}_{LCO},$$

where ${i}_{LCO}$ is the current density(in A m^−1^), $\nabla {\mathrm{\varnothing }}_{LCO}$ is the potential gradient, and ${\upsigma }_{LCO,eff}$ represents the effective electronic conductivity of LCO (in S m^−1^), which can be calculated as follows:

2 $${\upsigma }_{LCO,eff}= {\upvarepsilon }_{LCO}^{1.5}{\upsigma }_{LCO},$$

where ${\upsigma }_{LCO}$ is the electronic conductivity of LCO (in S m^−1^); ${\upvarepsilon }_{LCO}^{1.5}$ is Bruggeman factor, representing volume fraction and tortuosity of LCO in positive electrode.

*Li-ion diffusion in LCO* (LCO particles are considered to be perfect spheres surrounded by LLZO electrolyte):

3 $$\frac{\partial {c}_{{Li}^{+}}}{\partial t}=\nabla .\left({\mathrm{D}}_{{Li}^{+}}\nabla {c}_{{Li}^{+}}\right),$$

where ${c}_{{Li}^{+}}$ is the Li-ion concentration (in mol m^−3^), $t$ is time (in s) and ${\mathrm{D}}_{{Li}^{+}}$ is the intercalation diffusivity in LCO particles (in m^2^ s^−1^).

In order to solve the equation, the following two boundary conditions were applied:

4 $${\left.\frac{\partial {c}_{{Li}^{+}}}{\partial r}\right|}_{r=0}=0,$$

5 $${\left.-{D}_{{Li}^{+}}\frac{\partial {c}_{{Li}^{+}}}{\partial r}\right|}_{r={r}_{p}}=\sum_{m}\frac{{v}_{Li\theta ,m}{i}_{v,m}}{{n}_{m}F}\frac{{r}_{p}}{3{\upvarepsilon }_{LCO}},$$

where ${c}_{{Li}^{+}}$ is the species concentration (in mol m^−3^), ${r}_{p}$ is the particle mean center-surface distance (in m), ${D}_{{Li}^{+}}$ is the intercalation diffusivity constant (in m^2^ s^−1^), ${\upvarepsilon }_{LCO}$ is volume fraction of LCO in the electrode, ${v}_{Li\theta ,m}$ and ${n}_{m}$ are stoichiometric numbers of Li ions and electrons required for electrochemical lithiation/delithiation of LCO, $F$ is Faraday’s constant (96,485 C mol^−1^), and ${i}_{v,m}$ is the applied current (in A m^−2^).

The first condition (4) elucidates the particle diffusion rate at the center of LCO particles. The condition stems from the fact that the diffusion rate should be zero at the center of the sphere.

The second condition (5) implies the diffusion rate at the outer surface of spherical LCO particles. The diffusion rate at $r={r}_{p}$ is equalized to the reaction rate at the surface.

#### Li-ion transport in LLZO

The conduction of Li ions in the LLZO can be described by following generalized Ohm’s Law:

6 $${i}_{LLZO}=-{\upsigma }_{LLZO,eff}\nabla {\mathrm{\varnothing }}_{LLZO},$$

where ${i}_{LLZO}$ is the current density(in A/m), $\nabla {\mathrm{\varnothing }}_{LLZO}$ is the potential gradient, and ${\upsigma }_{LLZO,eff}$ represents the effective Li-ion conductivity of LLZO (in S m^−1^), which can be calculated as follows:

7 $${\upsigma }_{LLZO,eff}= {\upvarepsilon }_{LLZO}^{1.5}{\upsigma }_{LLZO},$$

where ${\upsigma }_{LLZO}$ is the Li-ion conductivity of LLZO (in S m^−1^); ${\upvarepsilon }_{LLZO}^{1.5}$ is Bruggeman factor, representing volume fraction and tortuosity of LLZO in positive electrode.

#### Butler–Volmer kinetics in positive electrode

8 $${i}_{loc,expr}={i}_{0} \left(exp\left(\frac{{\alpha }_{a}F\eta }{RT}\right)-exp\left(\frac{-{\alpha }_{c}F\eta }{RT}\right)\right),$$

where ${i}_{loc,expr}$ is the local exchange current density, ${\alpha }_{a}$ and ${\alpha }_{c}$ are the anodic and cathodic transfer coefficients, $\eta$ is the local over potential (in V), $T$ is the temperature (in K), and ${i}_{0}$ can be expressed as follows:

9 $${i}_{0}={i}_{0,ref}\left(T\right){\left(\frac{{c}_{{Li}^{+}}}{{c}_{{Li}^{+},ref}}\right)}^{{\alpha }_{c}}{\left(\frac{{{c}_{{Li}^{+},max}-c}_{{Li}^{+}}}{{c}_{{Li}^{+},max}-{c}_{{Li}^{+},ref}}\right)}^{{\alpha }_{a}},$$

10 $${ c}_{{Li}^{+},ref}=\frac{{c}_{{Li}^{+},max}}{2},$$

where ${c}_{{Li}^{+},ref}$ is reference lithium concentration of cathode at equilibrium state (in mol m^−3^), ${c}_{{Li}^{+},max}$ are reference and maximum lithium concentrations (in mol m^−3^), ${i}_{0,ref}$ is reference exchange current density of electrode (in A m^−2^), ${c}_{{Li}^{+}}$ is the lithium concentration on the surface of the active particles (in mol m^−3^).

*The initial charge of LCO* electrode (${Q}_{cell}$) was calculated as follows:

11 $${Q}_{cell}={c}_{{Li}^{+},max}\left({soc}_{max}-{soc}_{min}\right)\cdot F\cdot A\cdot {L}_{pos}\cdot {\upvarepsilon }_{LCO}$$

where ${c}_{{Li}^{+},max}$ is maximum lithium concentration, ${\upvarepsilon }_{LCO}$ is solid phase concentration, ${soc}_{max}$ and ${soc}_{min}$ are maximum and minimum state of charge and ${L}_{pos}$ is the thickness of the positive electrode. Apart from them, $F$ and $A$ are Faraday’s constant (96,485 C mol^−1^), and area of the cell (in m^2^), respectively.

### Simulations of Li negative electrode

*The electronic conductivity* in Li negative electrode is governed by generalized Ohm’s Law as indicated above for LCO cathode.

*Butler–Volmer kinetics* is described as follows:

12 $${i}_{loc,expr}={i}_{0} \left(exp\left(\frac{{\alpha }_{a}F\eta }{RT}\right)-exp\left(\frac{-{\alpha }_{c}F\eta }{RT}\right)\right),$$

where $${i}_{0}={i}_{0,ref}$$.

### Simulations of LLZO solid-state electrolyte

The electrolyte was modeled as single-ion conductor, considering solely the ionic conductivity of electrolyte while calculating the charge. Temperature dependence of Li-ion conductivity of LLZO is considered while performing simulations at different temperatures.

The conduction of Li ions in the LLZO solid-state electrolyte is described by following generalized Ohm’s Law:

13 $${i}_{LLZO}=-{\upsigma }_{LLZO}\nabla {\mathrm{\varnothing }}_{LLZO},$$

where ${\upsigma }_{LLZO}$ is the ionic conductivity of LLZO (in S m^−1^) and $\nabla {\mathrm{\varnothing }}_{LLZO}$ is electrolyte phase potential gradient.

## Supplementary Information

Supplementary Information.
