# Supplementary material for: On the feasibility of all-solid-state batteries with LLZO as a single electrolyte
Source: Sci Rep. 2022 Jan 21;12:1177. doi: 10.1038/s41598-022-05141-x (PMC8782839; doi:10.1038/s41598-022-05141-x)
Supplement: Supplementary file 1 — Supplementary Information. [file 41598_2022_5141_MOESM1_ESM.docx]

*Supporting Information for*

On the feasibility of all-solid-state batteries with LLZO as a single electrolyte

Kostiantyn V. Kravchyk,^1,2^ Dogan Tarik Karabay^1,2^ and Maksym V. Kovalenko^1,2*^

^1^ Laboratory for Thin Films and Photovoltaics, Empa – Swiss Federal Laboratories for Materials Science and Technology, Überlandstrasse 129, CH-8600 Dübendorf, Switzerland

^2^ Laboratory of Inorganic Chemistry, Department of Chemistry and Applied Biosciences, ETH Zürich, Vladimir-Prelog-Weg 1, CH-8093 Zürich, Switzerland

Corresponding Authors:

*E-mails: [kravchyk@inorg.chem.ethz.ch](mailto:kravchyk@inorg.chem.ethz.ch) and [mvkovalenko@ethz.ch](mailto:mvkovalenko@ethz.ch)

**
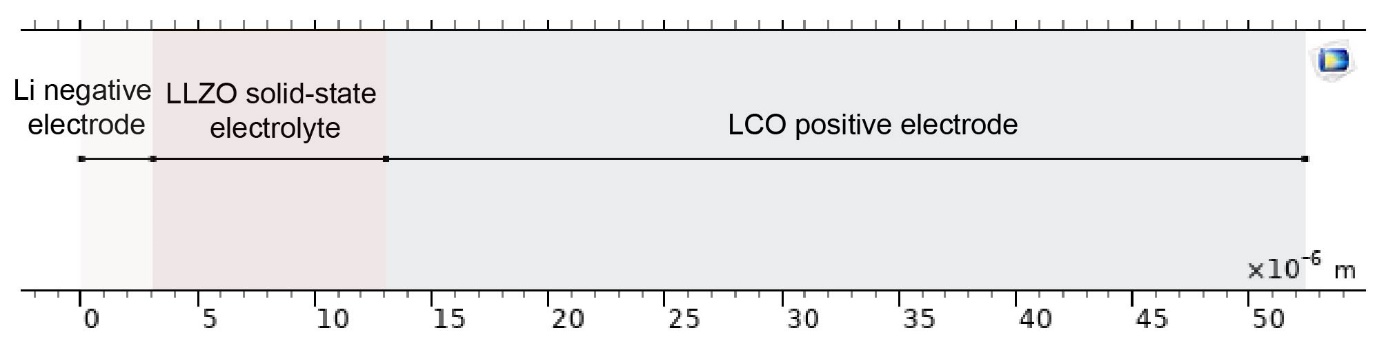
**

**Figure S1.** Schematic of the model used for simulations.

**
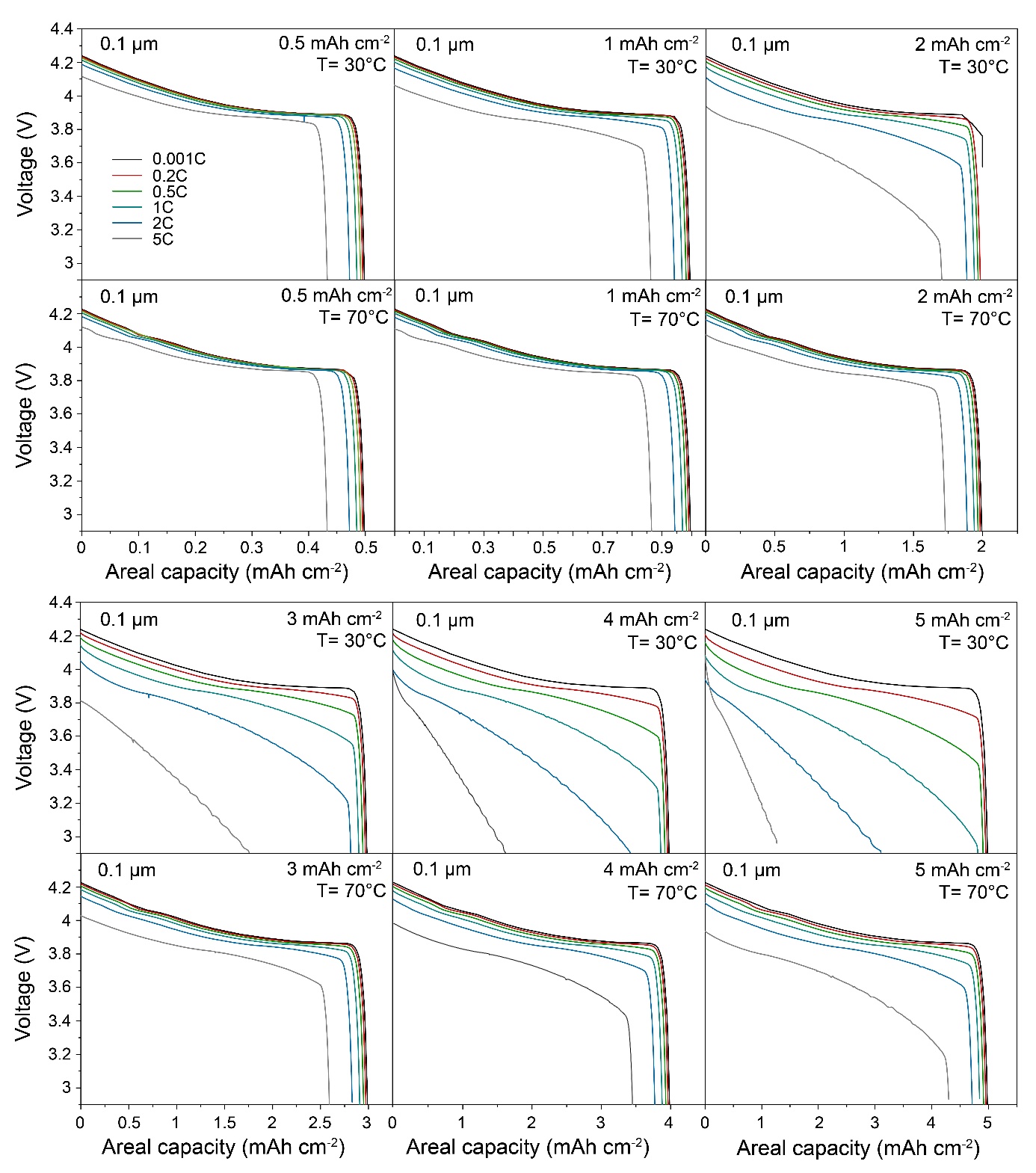
**

**Figures S2.** Simulated voltage profiles of Li/LLZO/LCO all-solid-state battery at different cathode areal capacities (0.5, 1, 2, 3, 4, and 5 mAh cm^-2^), C rates (0.001C, 0.2C, 0.5C, 1C, 2C, and 5C) and temperatures (30°C and 70°C). LLZO thickness is constant of 0.1 µm. LCO cathode is composed of 70 vol.% of LCO and 30 vol.% of LLZO.

**
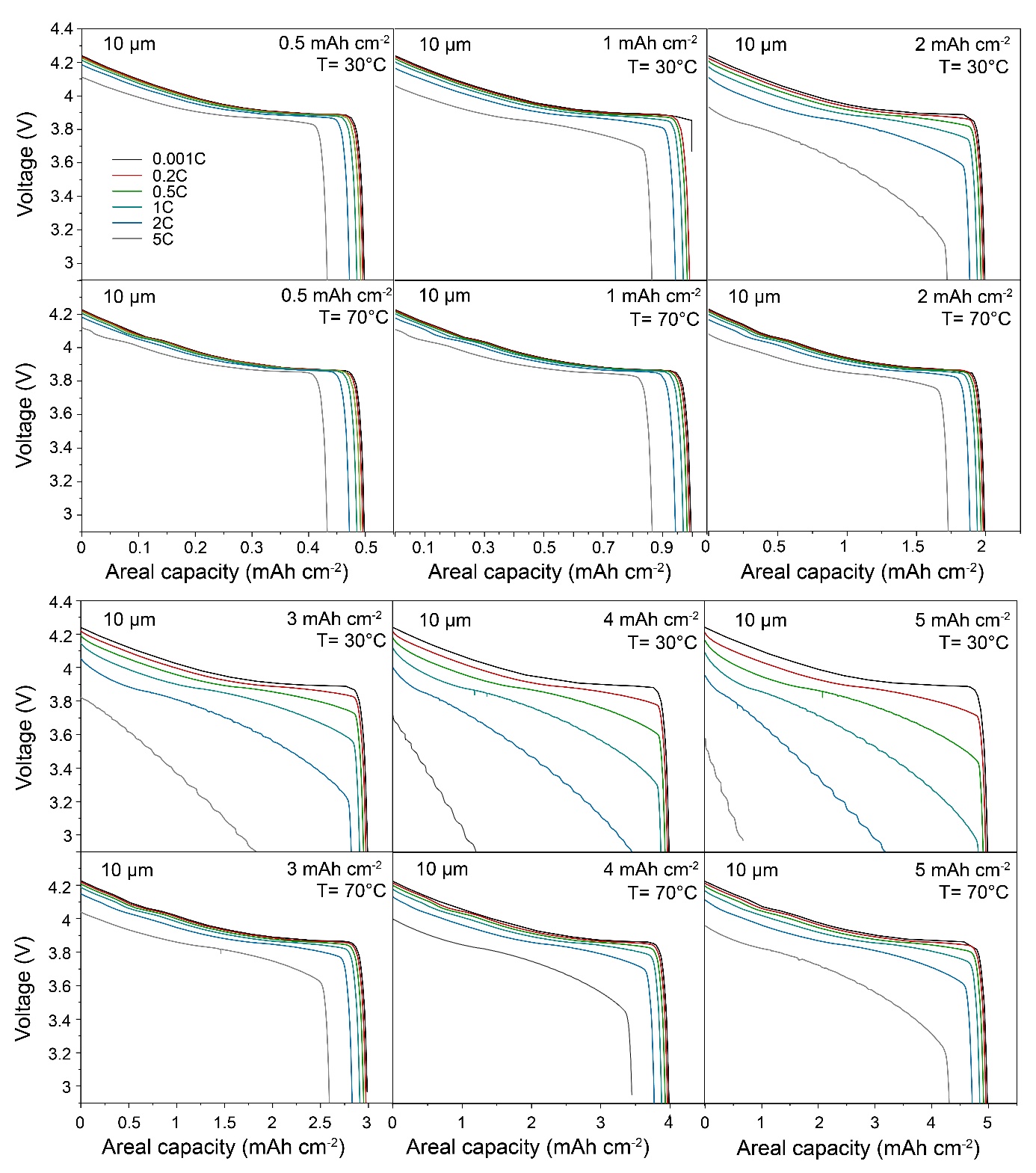
**

**Figures S3.** Simulated voltage profiles of Li/LLZO/LCO all-solid-state battery at different cathode areal capacities (0.5, 1, 2, 3, 4, and 5 mAh cm^-2^), C rates (0.001C, 0.2C, 0.5C, 1C, 2C, and 5C) and temperatures (30°C and 70°C). LLZO thickness is constant of 10 µm. LCO cathode is composed of 70 vol.% of LCO and 30 vol.% of LLZO.

**
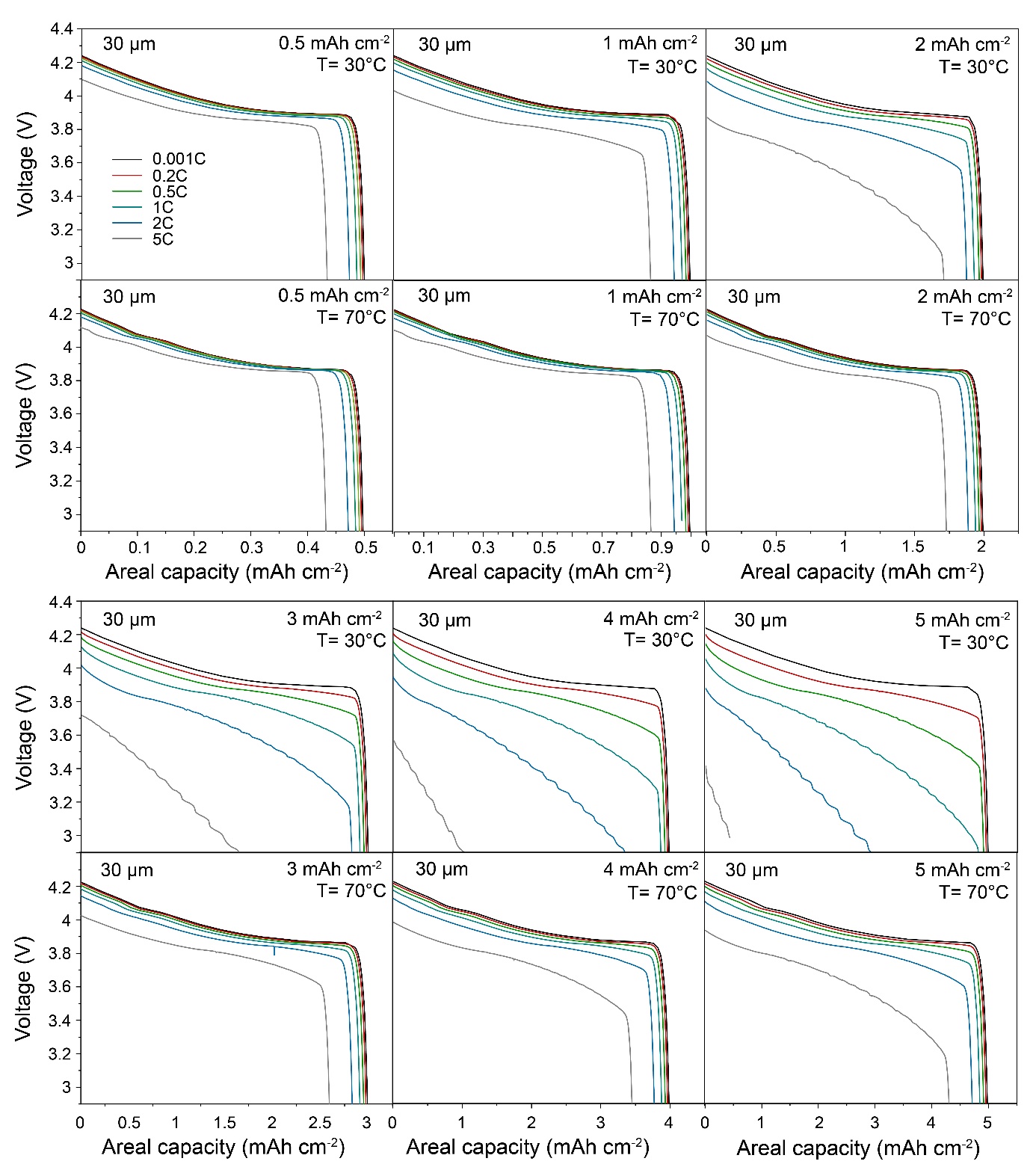
**

**Figures S4.** Simulated voltage profiles of Li/LLZO/LCO all-solid-state battery at different cathode areal capacities (0.5, 1, 2, 3, 4, and 5 mAh cm^-2^), C rates (0.001C, 0.2C, 0.5C, 1C, 2C, and 5C) and temperatures (30°C and 70°C). LLZO thickness is constant of 30 µm. LCO cathode is composed of 70 vol.% of LCO and 30 vol.% of LLZO.

**
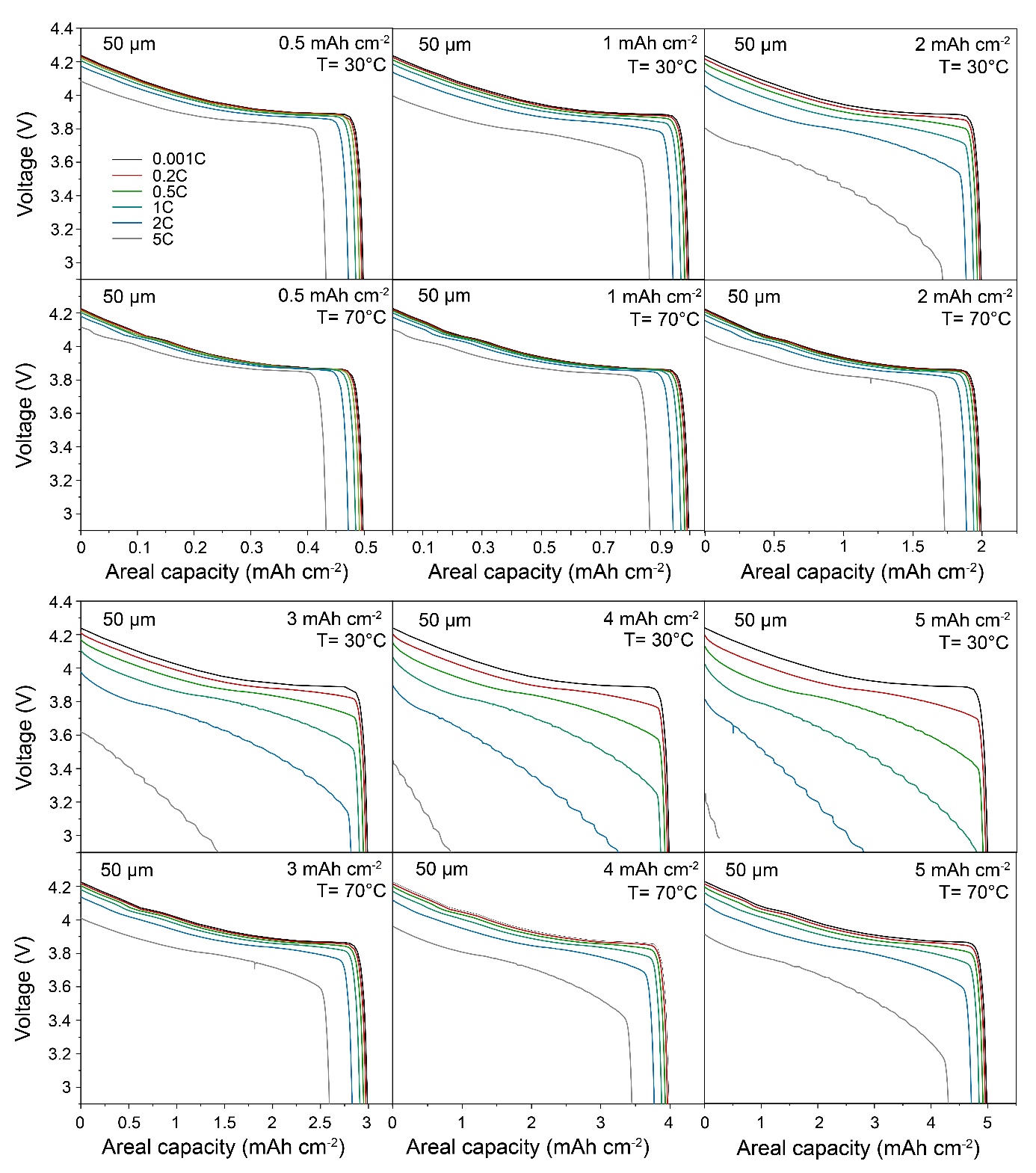
**

**Figures S5.** Simulated voltage profiles of Li/LLZO/LCO all-solid-state battery at different cathode areal capacities (0.5, 1, 2, 3, 4, and 5 mAh cm^-2^), C rates (0.001C, 0.2C, 0.5C, 1C, 2C, and 5C) and temperatures (30°C and 70°C). LLZO thickness is constant of 50 µm. LCO cathode is composed of 70 vol.% of LCO and 30 vol.% of LLZO.

**
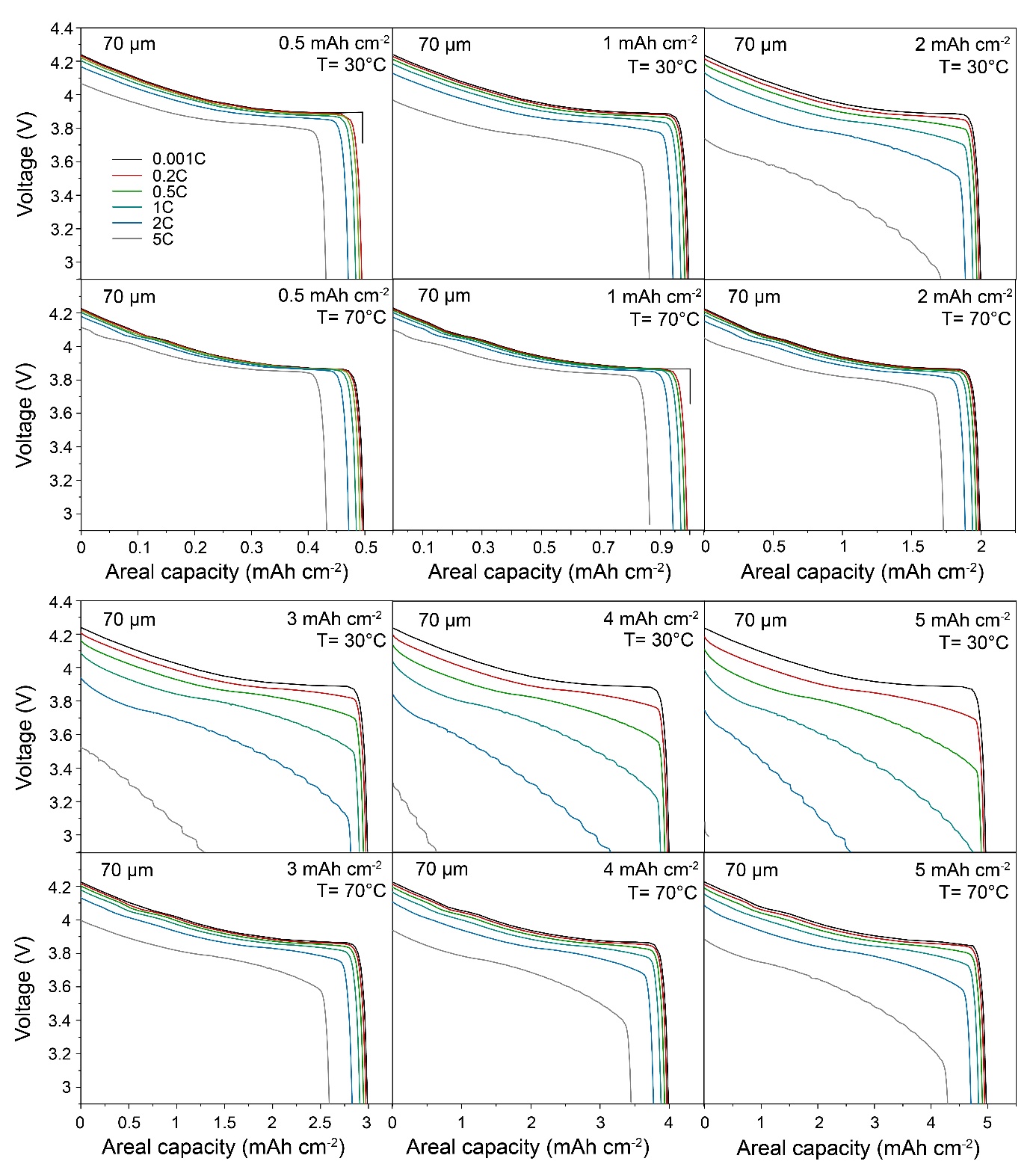
**

**Figures S6.** Simulated voltage profiles of Li/LLZO/LCO all-solid-state battery at different cathode areal capacities (0.5, 1, 2, 3, 4, and 5 mAh cm^-2^), C rates (0.001C, 0.2C, 0.5C, 1C, 2C, and 5C) and temperatures (30°C and 70°C). LLZO thickness is constant of 70 µm. LCO cathode is composed of 70 vol.% of LCO and 30 vol.% of LLZO.

**
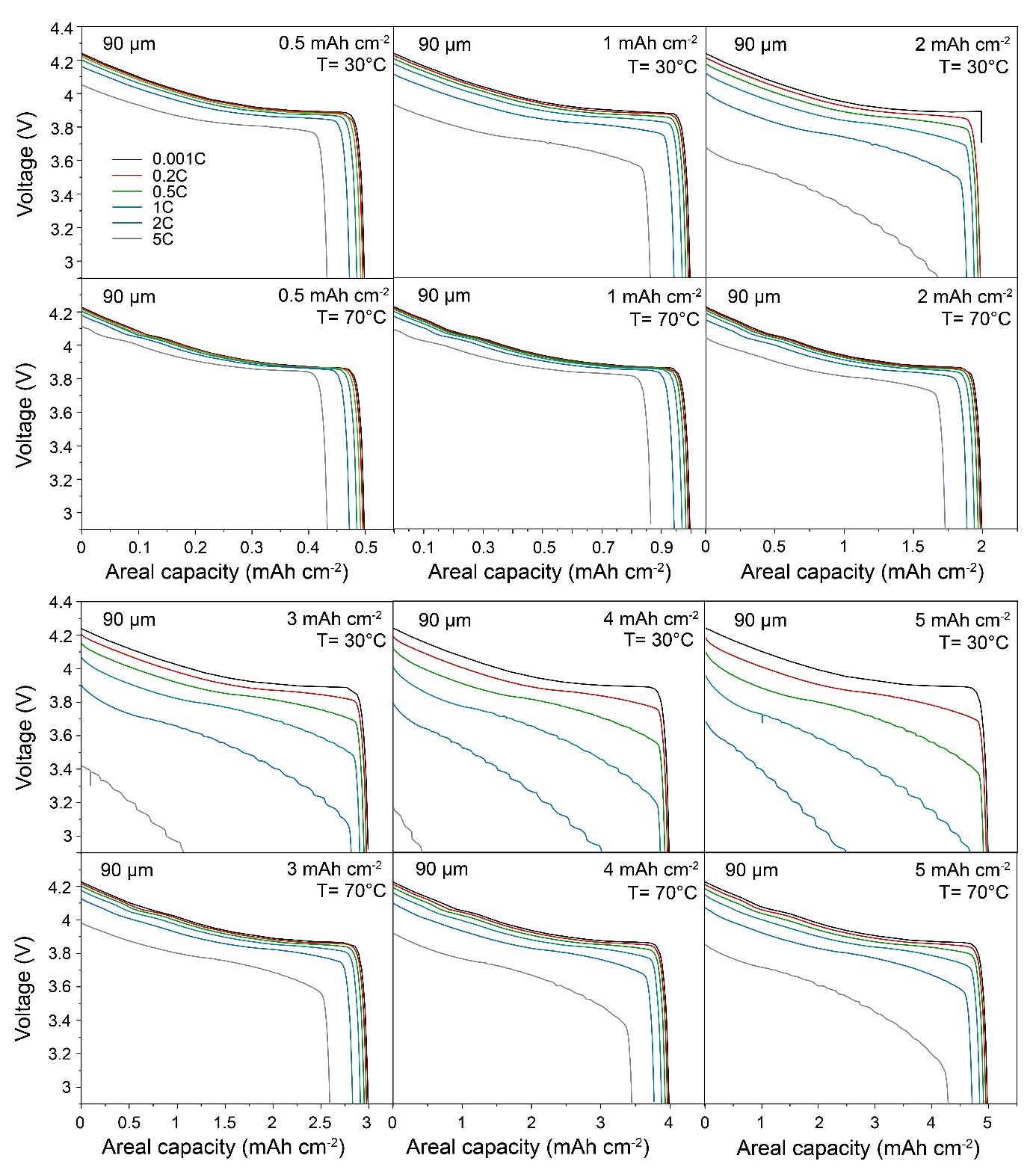
**

**Figures S7.** Simulated voltage profiles of Li/LLZO/LCO all-solid-state battery at different cathode areal capacities (0.5, 1, 2, 3, 4, and 5 mAh cm^-2^), C rates (0.001C, 0.2C, 0.5C, 1C, 2C, and 5C) and temperatures (30°C and 70°C). LLZO thickness is constant of 90 µm. LCO cathode is composed of 70 vol.% of LCO and 30 vol.% of LLZO.

**
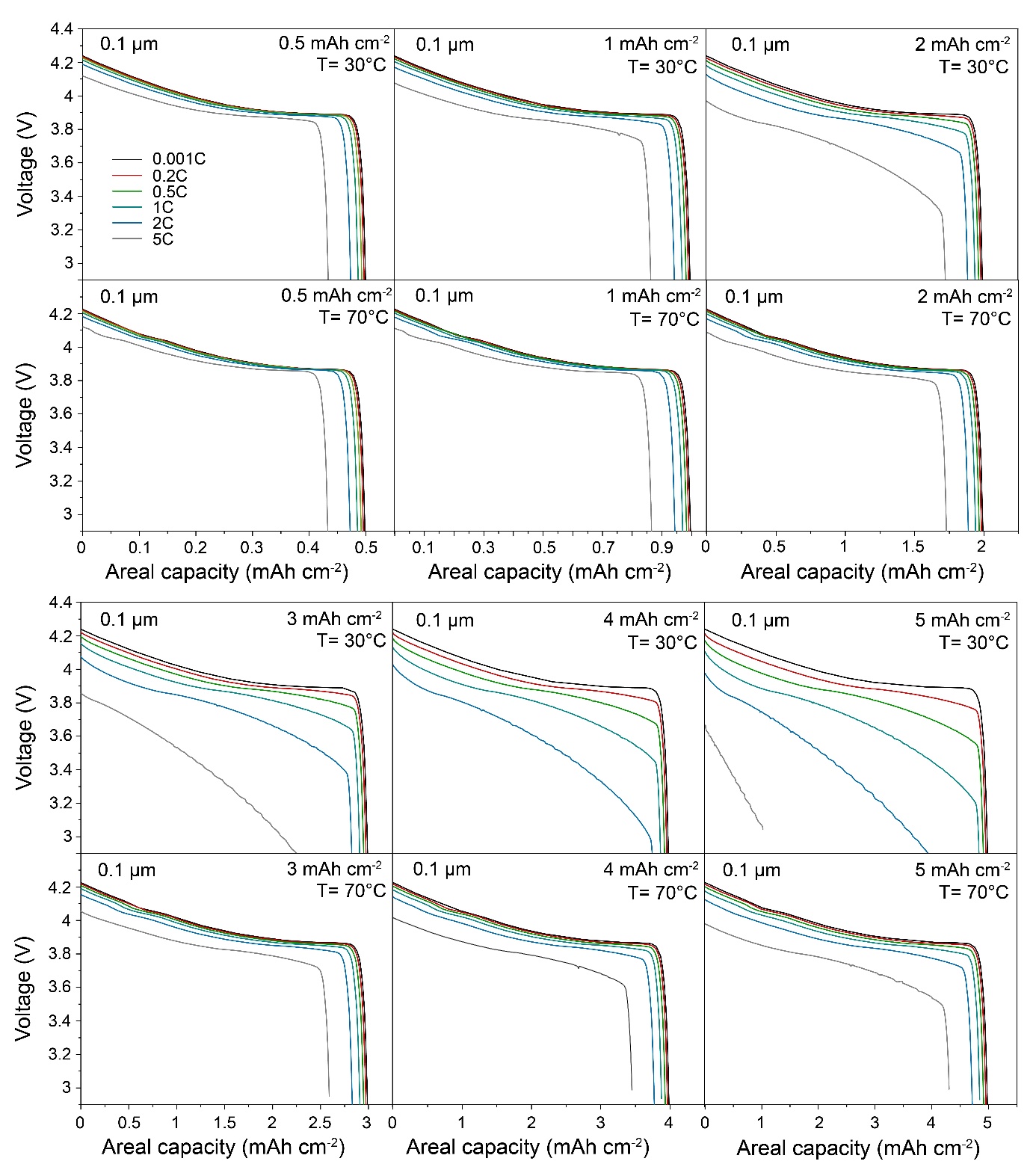

Figures S8.** Simulated voltage profiles of Li/LLZO/LCO all-solid-state battery at different cathode areal capacities (0.5, 1, 2, 3, 4, and 5 mAh cm^-2^), C rates (0.001C, 0.2C, 0.5C, 1C, 2C, and 5C) and temperatures (30°C and 70°C). LLZO thickness is constant of 0.1 µm. LCO cathode is composed of 60 vol.% of LCO and 40 vol.% of LLZO.

**
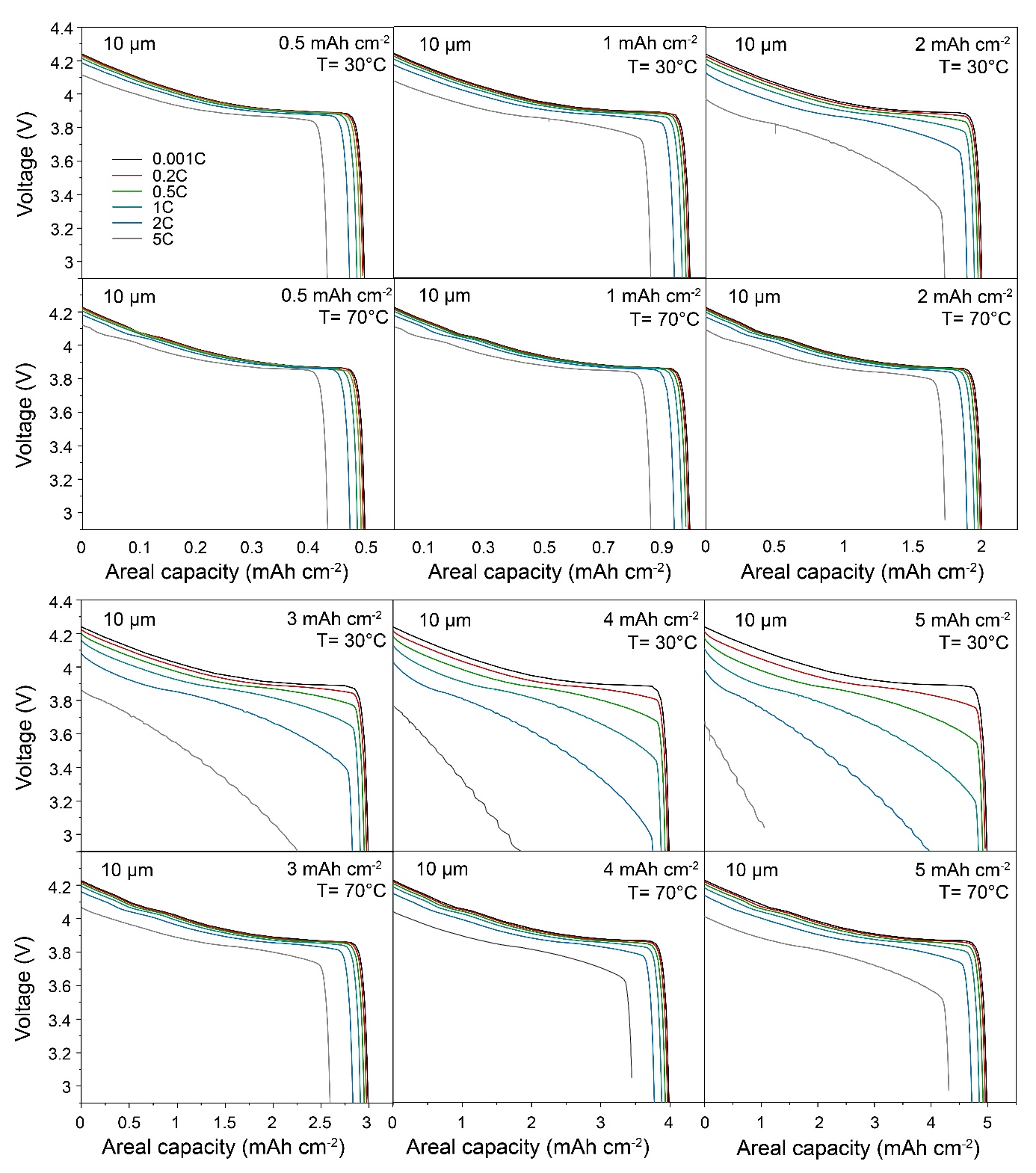
**

**Figures S9.** Simulated voltage profiles of Li/LLZO/LCO all-solid-state battery at different cathode areal capacities (0.5, 1, 2, 3, 4, and 5 mAh cm^-2^), C rates (0.001C, 0.2C, 0.5C, 1C, 2C, and 5C) and temperatures (30°C and 70°C). LLZO thickness is constant of 10 µm. LCO cathode is composed of 60 vol.% of LCO and 40 vol.% of LLZO.

**
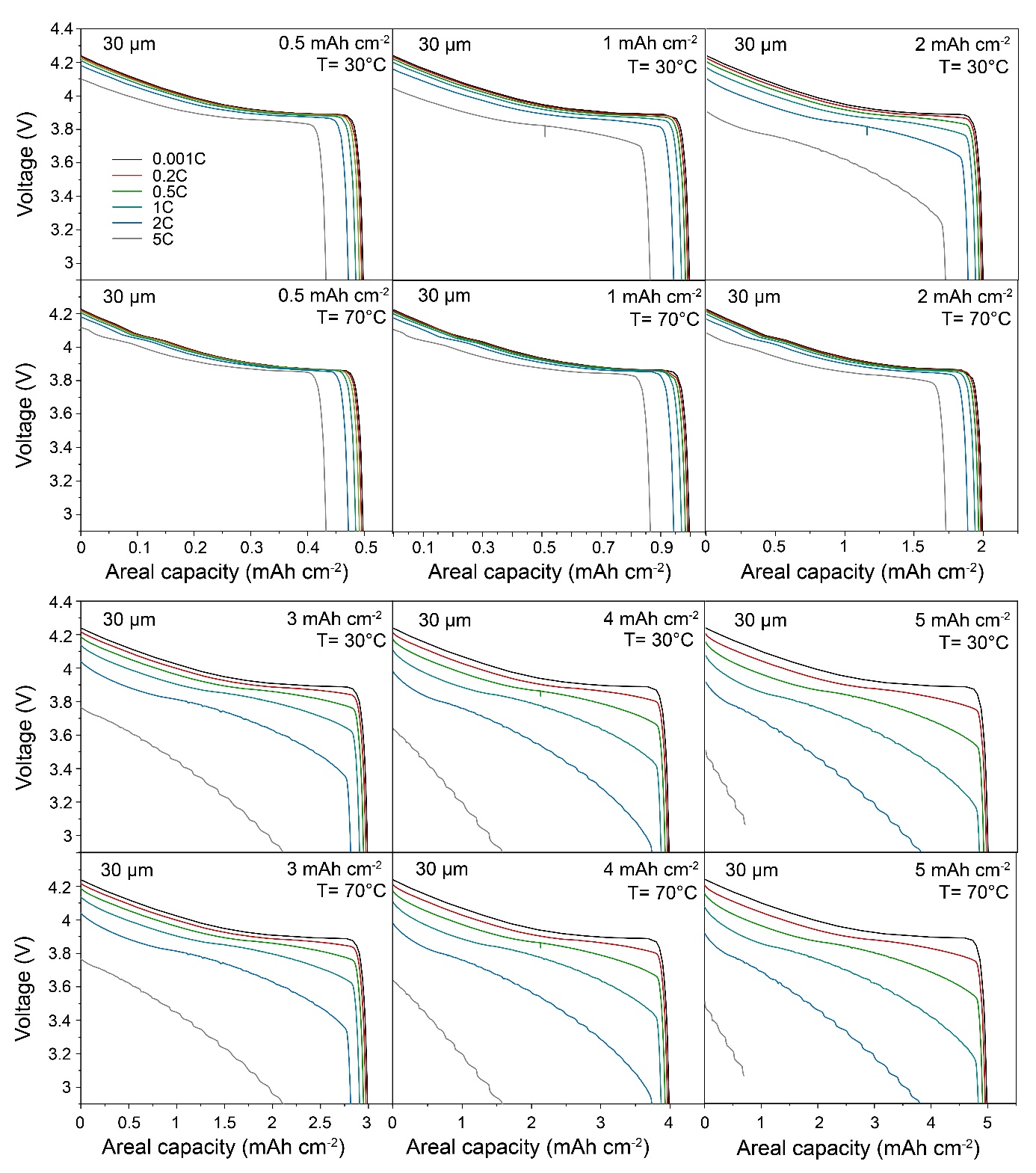
**

**Figures S10.** Simulated voltage profiles of Li/LLZO/LCO all-solid-state battery at different cathode areal capacities (0.5, 1, 2, 3, 4, and 5 mAh cm^-2^), C rates (0.001C, 0.2C, 0.5C, 1C, 2C, and 5C) and temperatures (30°C and 70°C). LLZO thickness is constant of 30 µm. LCO cathode is composed of 60 vol.% of LCO and 40 vol.% of LLZO.

**
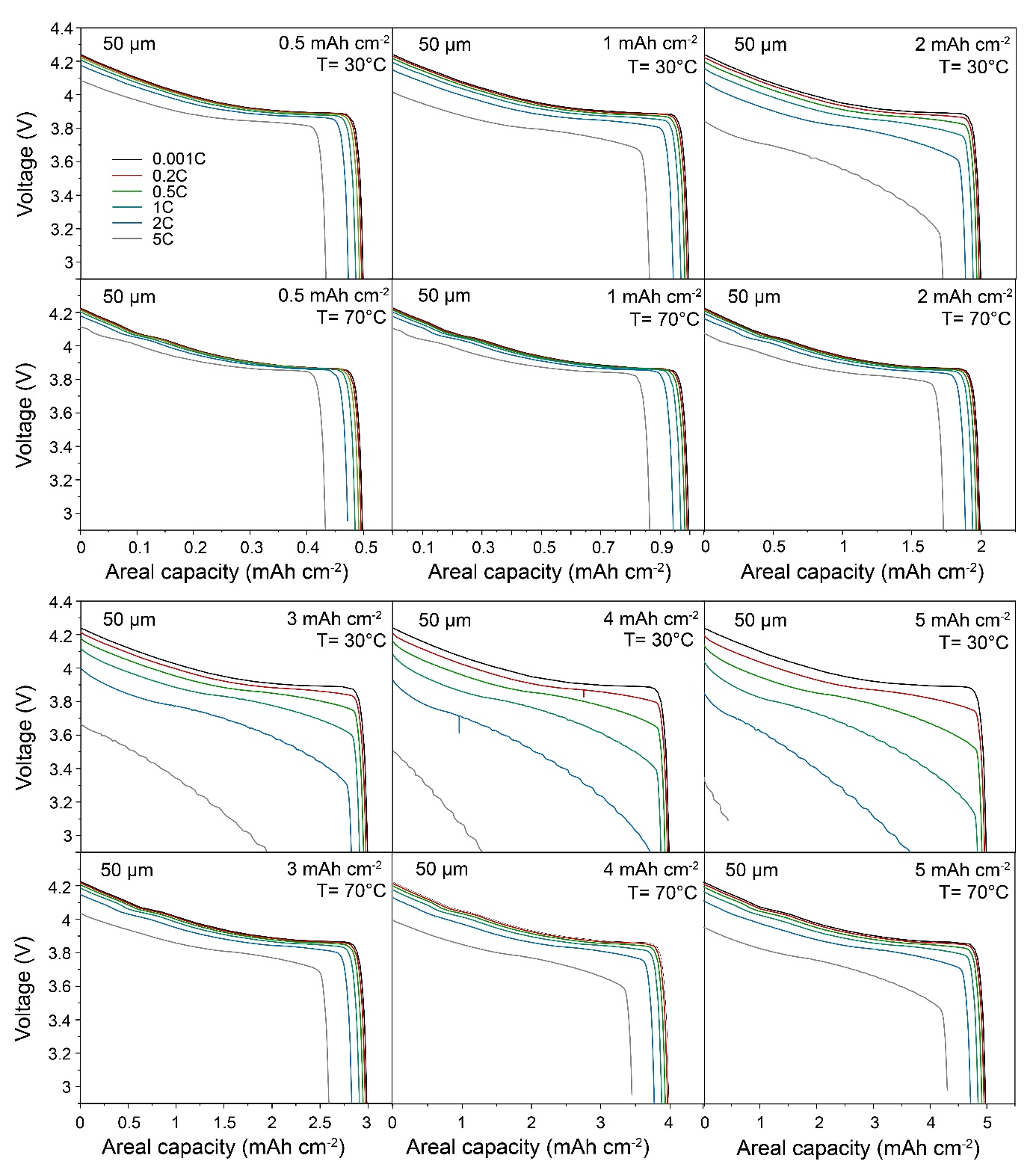
**

**Figures S11.** Simulated voltage profiles of Li/LLZO/LCO all-solid-state battery at different cathode areal capacities (0.5, 1, 2, 3, 4, and 5 mAh cm^-2^), C rates (0.001C, 0.2C, 0.5C, 1C, 2C, and 5C) and temperatures (30°C and 70°C). LLZO thickness is constant of 50 µm. LCO cathode is composed of 60 vol.% of LCO and 40 vol.% of LLZO.

**
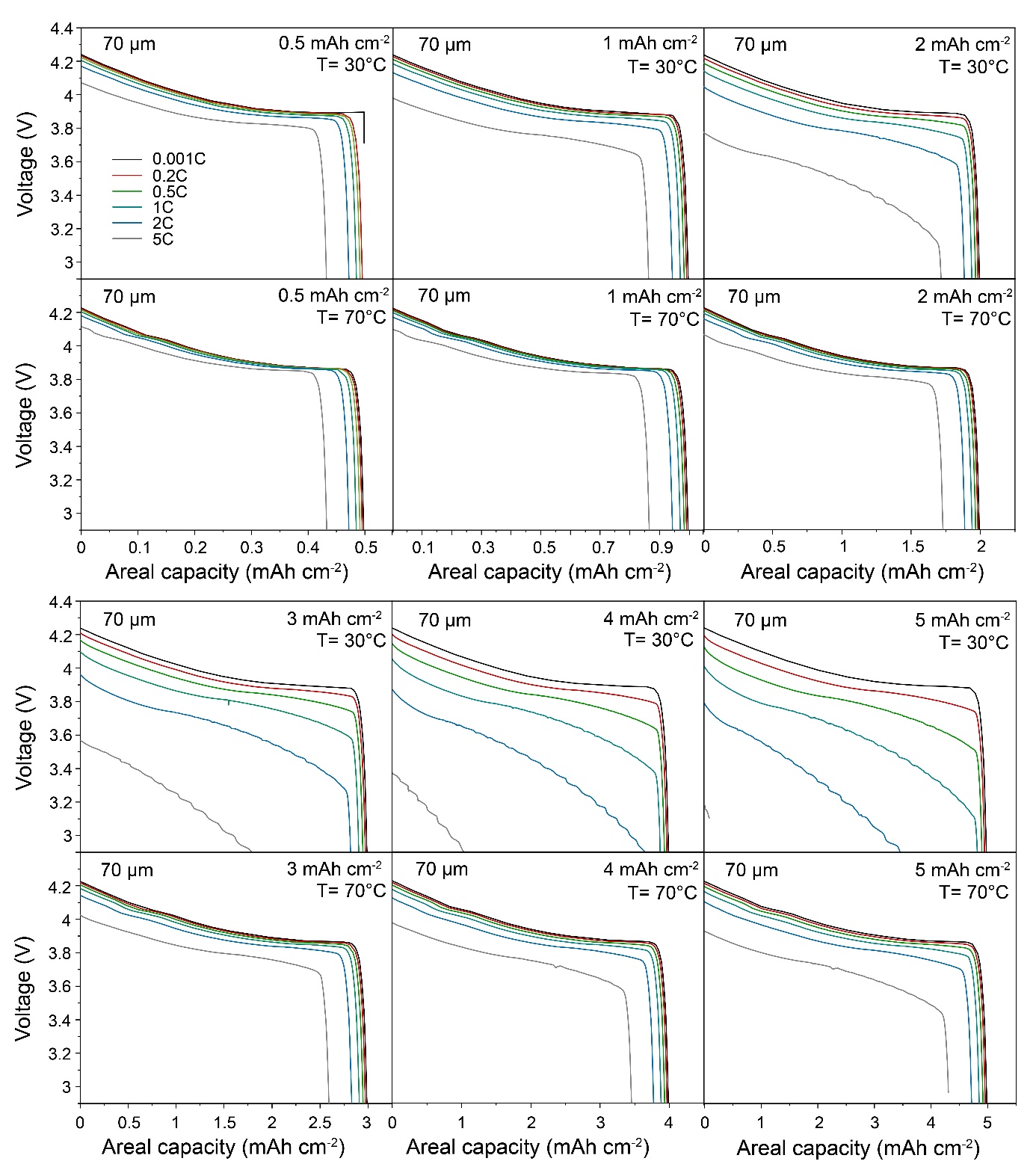
**

**Figures S12.** Simulated voltage profiles of Li/LLZO/LCO all-solid-state battery at different cathode areal capacities (0.5, 1, 2, 3, 4, and 5 mAh cm^-2^), C rates (0.001C, 0.2C, 0.5C, 1C, 2C, and 5C) and temperatures (30°C and 70°C). LLZO thickness is constant of 70 µm. LCO cathode is composed of 60 vol.% of LCO and 40 vol.% of LLZO.

**
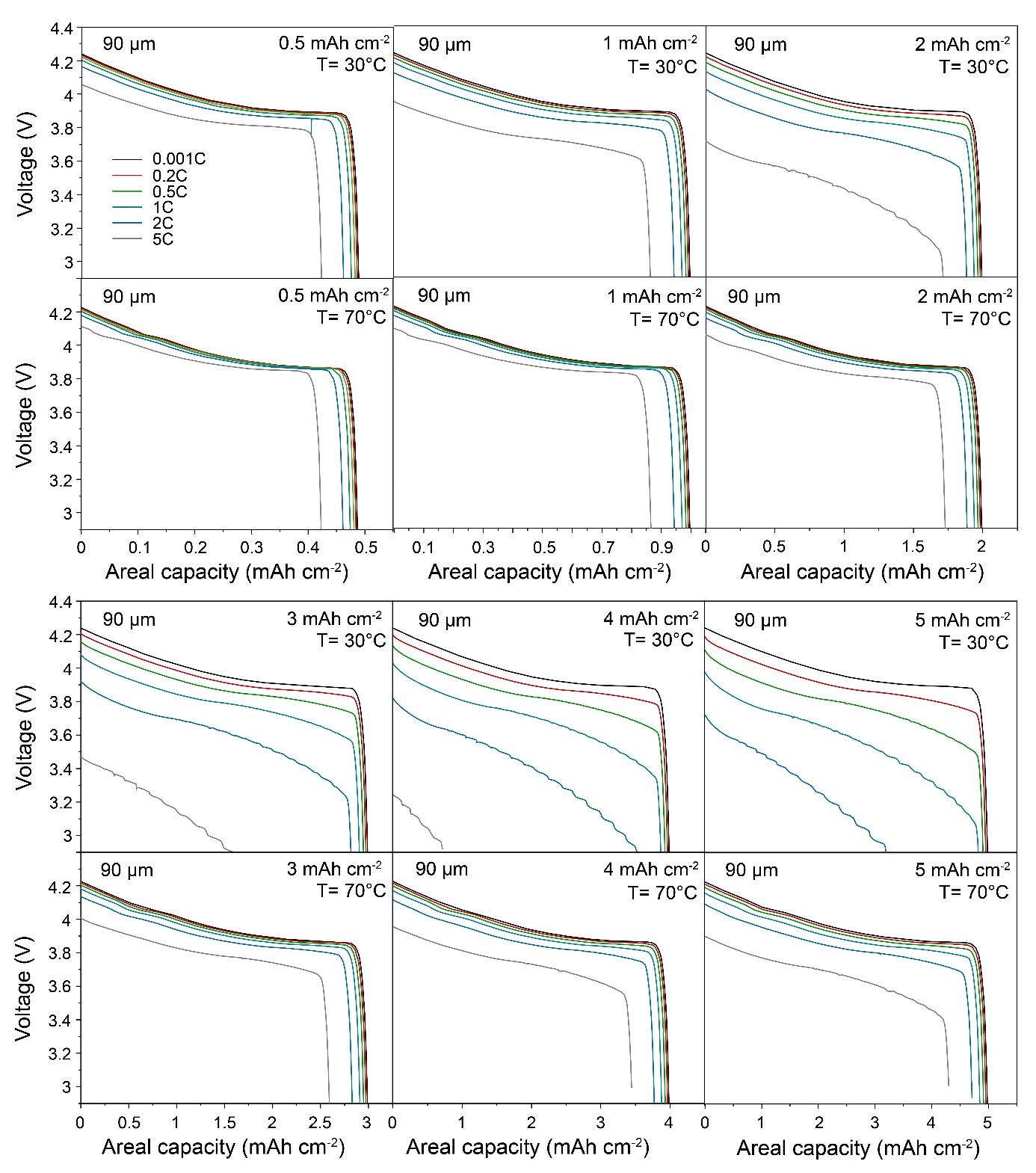
**

**Figures S13.** Simulated voltage profiles of Li/LLZO/LCO all-solid-state battery at different cathode areal capacities (0.5, 1, 2, 3, 4, and 5 mAh cm^-2^), C rates (0.001C, 0.2C, 0.5C, 1C, 2C, and 5C) and temperatures (30°C and 70°C). LLZO thickness is constant of 90 µm. LCO cathode is composed of 60 vol.% of LCO and 40 vol.% of LLZO.

**
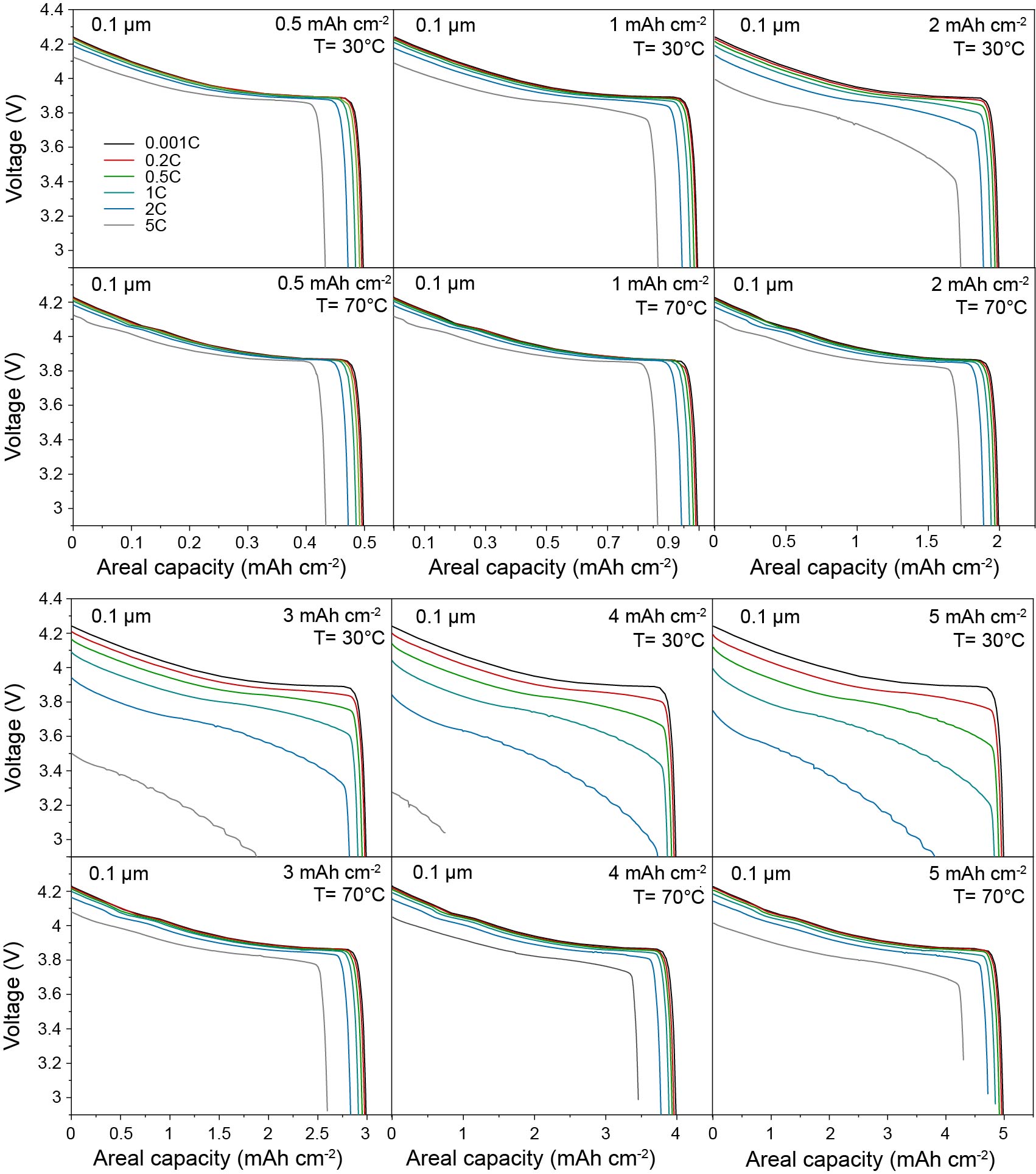
**

**Figures S14.** Simulated voltage profiles of Li/LLZO/LCO all-solid-state battery at different cathode areal capacities (0.5, 1, 2, 3, 4, and 5 mAh cm^-2^), C rates (0.001C, 0.2C, 0.5C, 1C, 2C, and 5C) and temperatures (30°C and 70°C). LLZO thickness is constant of 0.1 µm. LCO cathode is composed of 50 vol.% of LCO and 50 vol.% of LLZO.

**
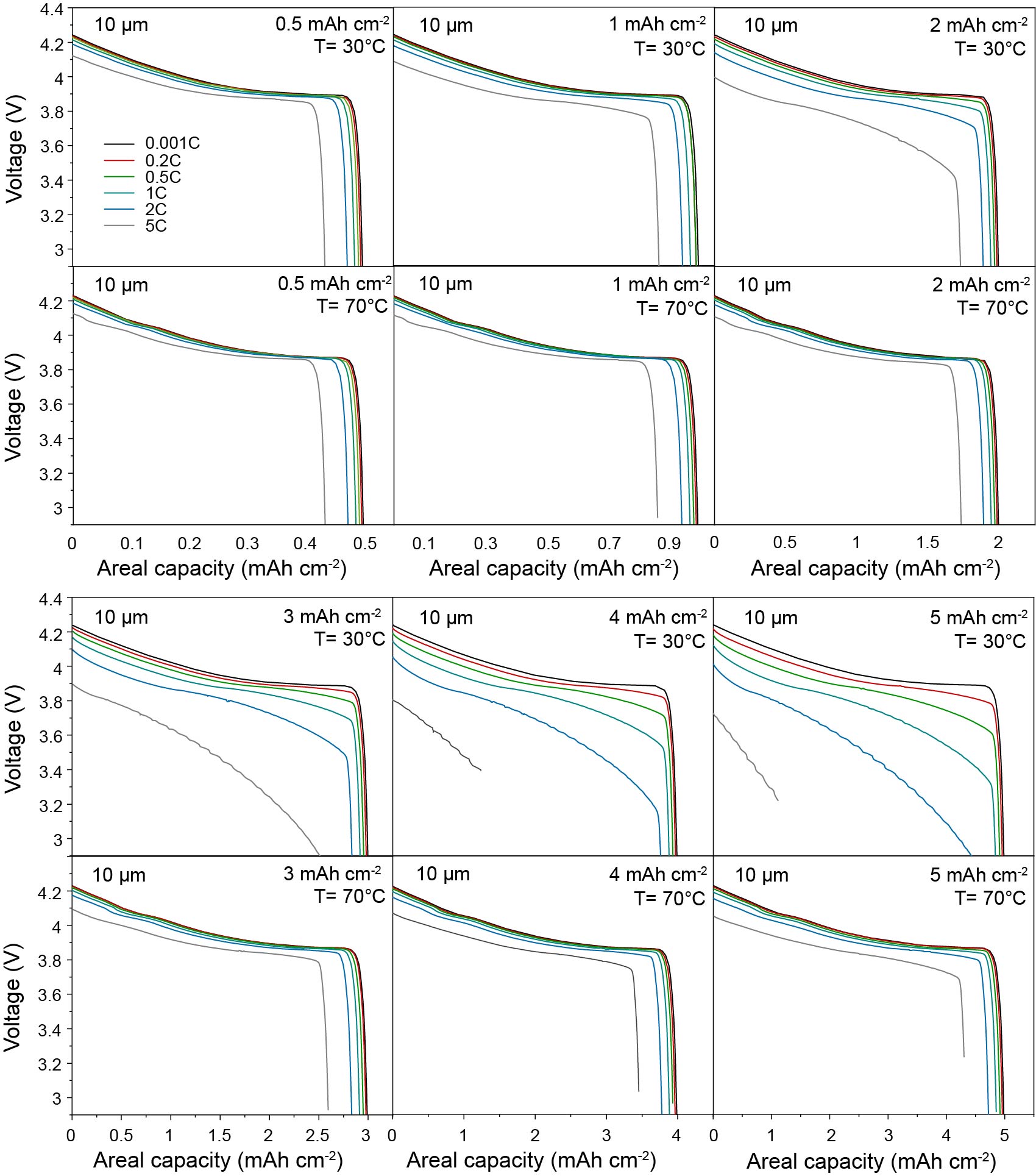
**

**Figures S15.** Simulated voltage profiles of Li/LLZO/LCO all-solid-state battery at different cathode areal capacities (0.5, 1, 2, 3, 4, and 5 mAh cm^-2^), C rates (0.001C, 0.2C, 0.5C, 1C, 2C, and 5C) and temperatures (30°C and 70°C). LLZO thickness is constant of 10 µm. LCO cathode is composed of 50 vol.% of LCO and 50 vol.% of LLZO.

**
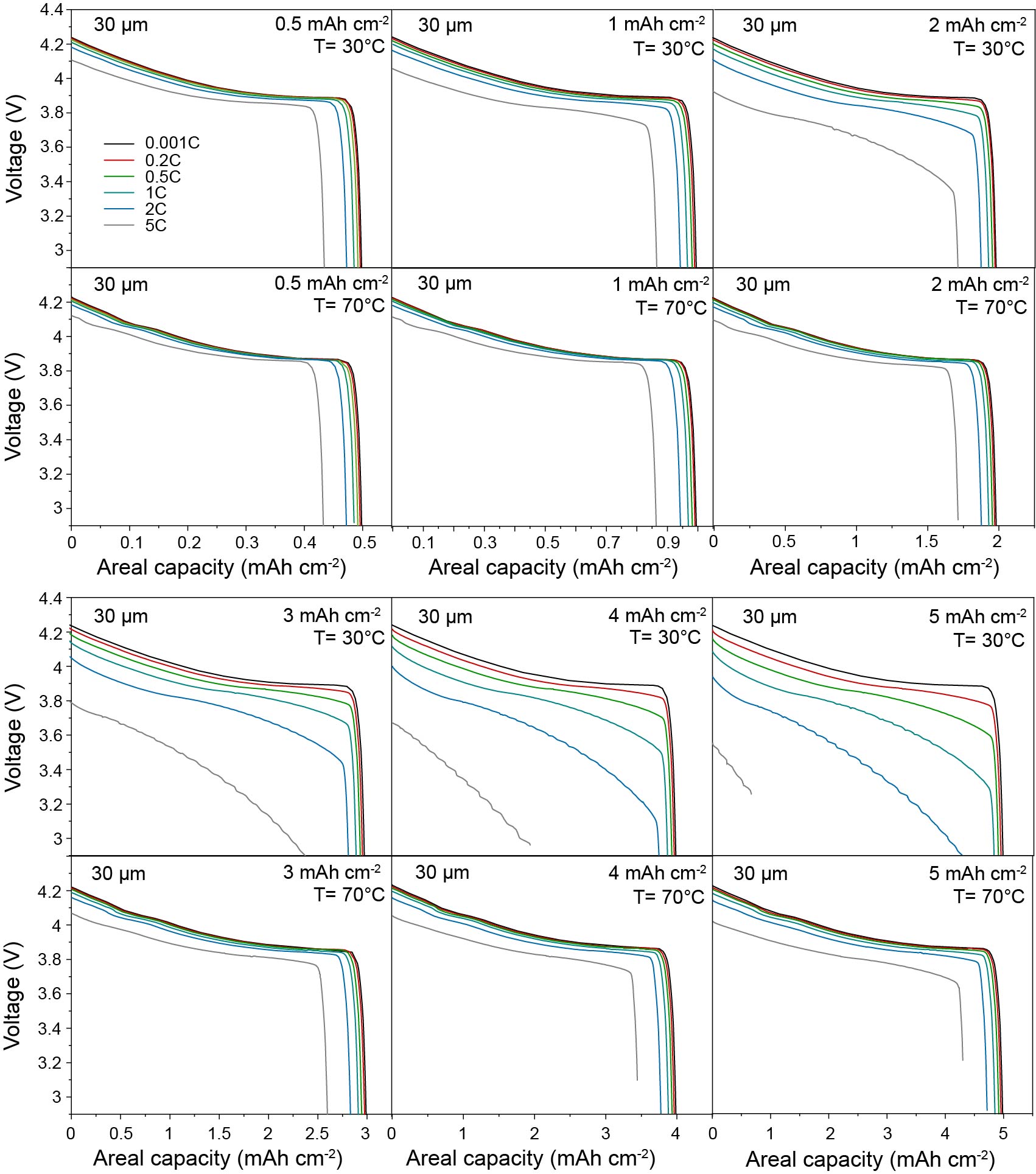
**

**Figures S16.** Simulated voltage profiles of Li/LLZO/LCO all-solid-state battery at different cathode areal capacities (0.5, 1, 2, 3, 4, and 5 mAh cm^-2^), C rates (0.001C, 0.2C, 0.5C, 1C, 2C, and 5C) and temperatures (30°C and 70°C). LLZO thickness is constant of 30 µm. LCO cathode is composed of 50 vol.% of LCO and 50 vol.% of LLZO.

**
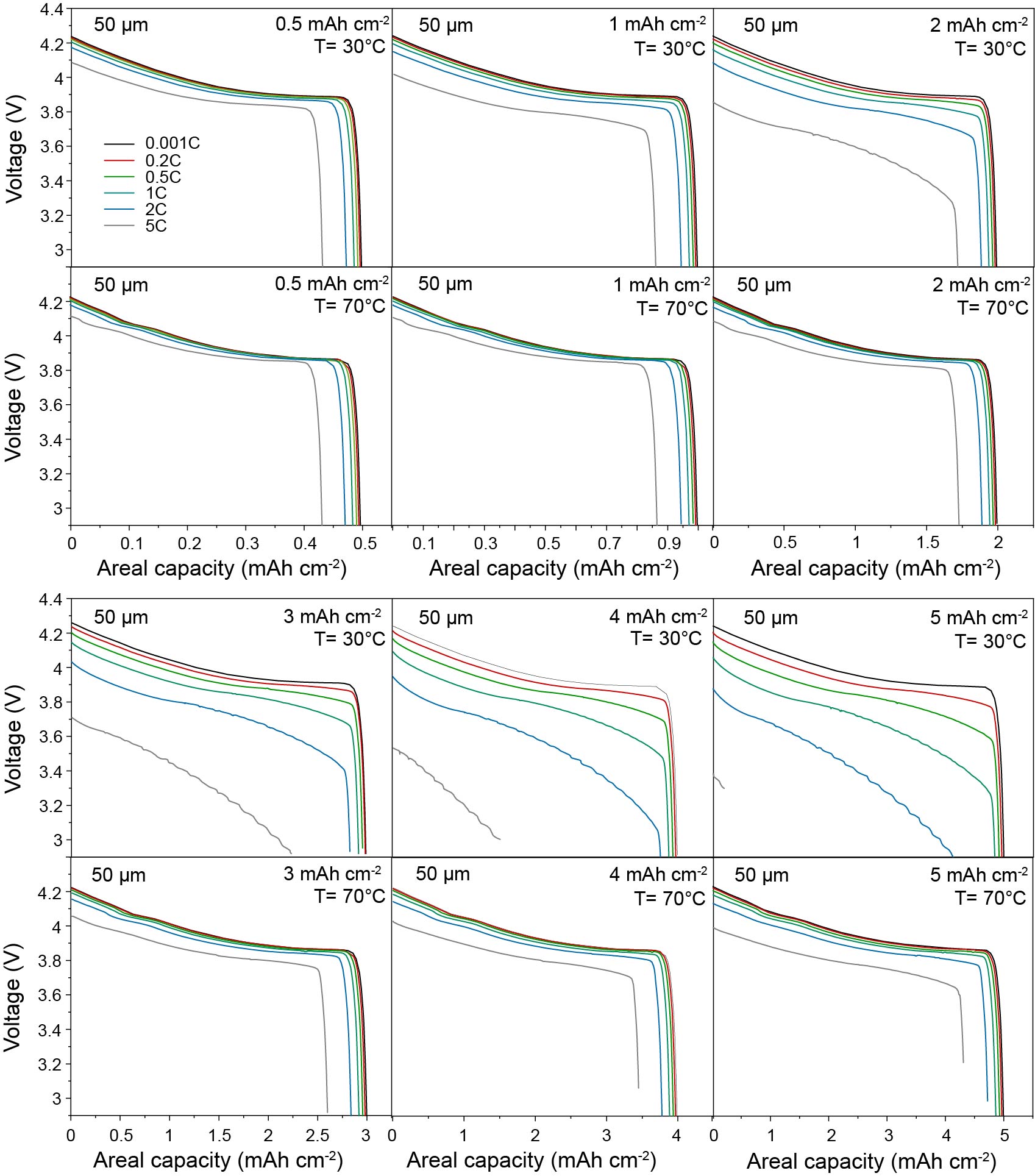
**

**Figures S17.** Simulated voltage profiles of Li/LLZO/LCO all-solid-state battery at different cathode areal capacities (0.5, 1, 2, 3, 4, and 5 mAh cm^-2^), C rates (0.001C, 0.2C, 0.5C, 1C, 2C, and 5C) and temperatures (30°C and 70°C). LLZO thickness is constant of 50 µm. LCO cathode is composed of 50 vol.% of LCO and 50 vol.% of LLZO.

**
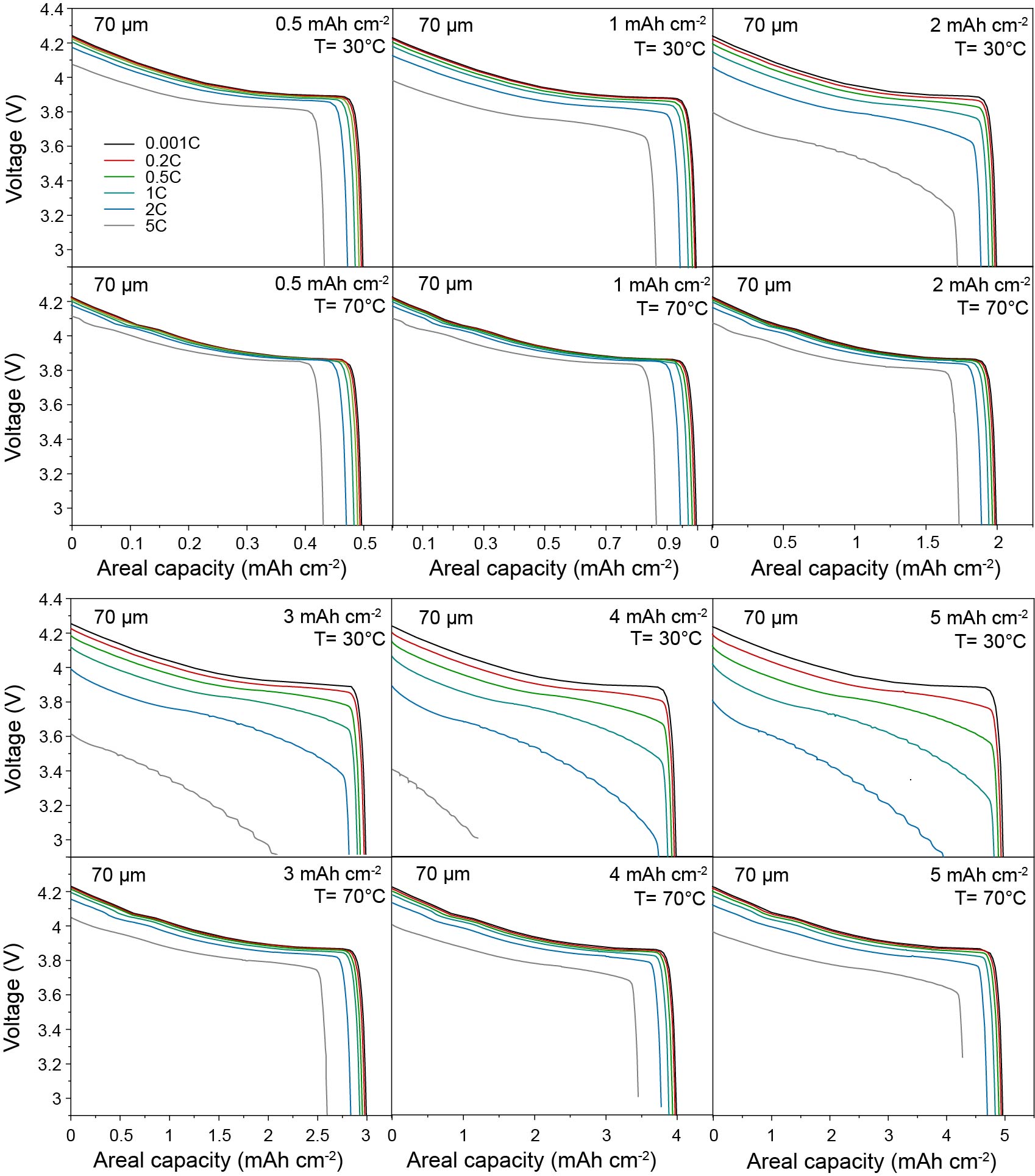
**

**Figures S18.** Simulated voltage profiles of Li/LLZO/LCO all-solid-state battery at different cathode areal capacities (0.5, 1, 2, 3, 4, and 5 mAh cm^-2^), C rates (0.001C, 0.2C, 0.5C, 1C, 2C, and 5C) and temperatures (30°C and 70°C). LLZO thickness is constant of 70 µm. LCO cathode is composed of 50 vol.% of LCO and 50 vol.% of LLZO.

**
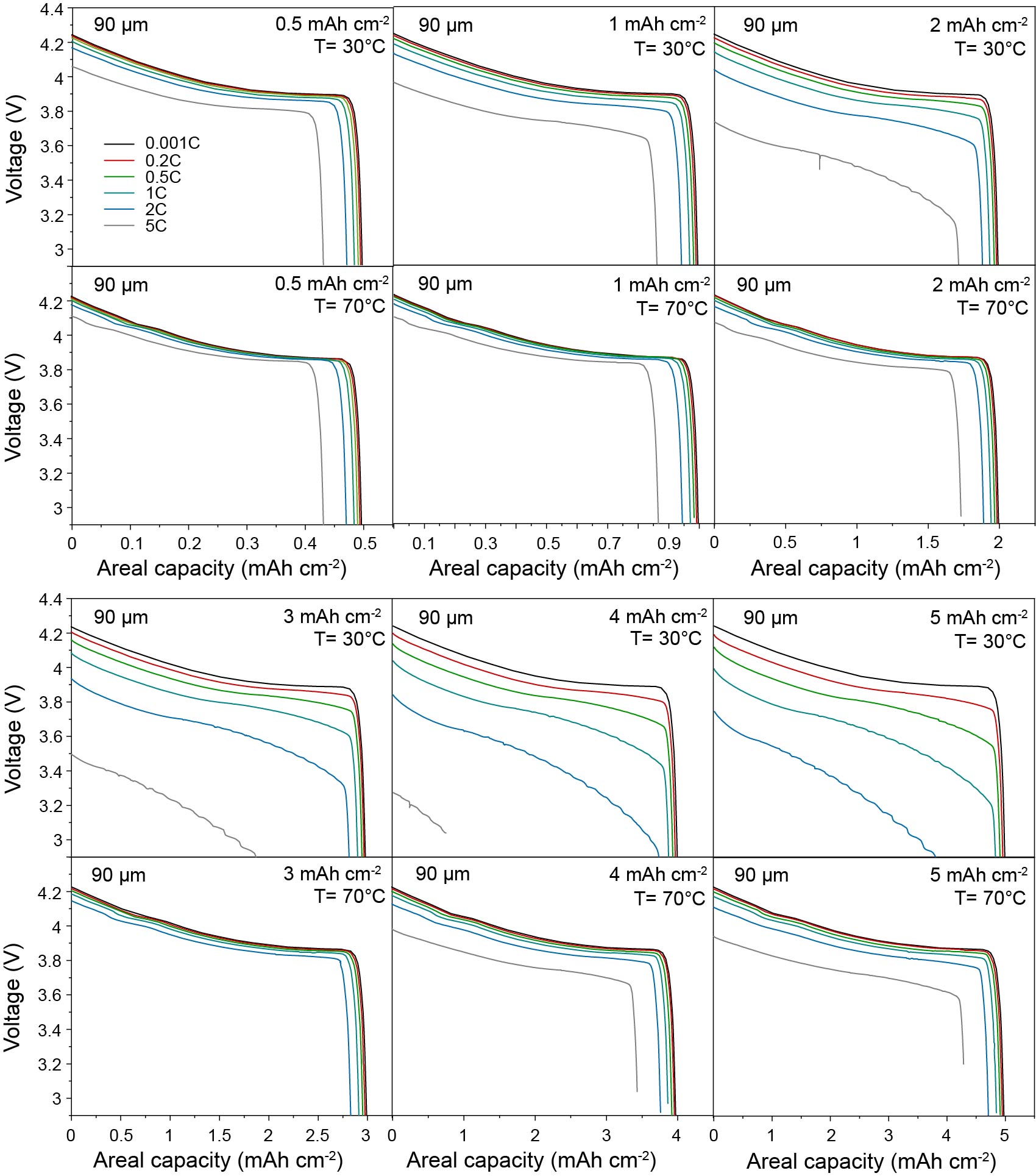
**

**Figures S19.** Simulated voltage profiles of Li/LLZO/LCO all-solid-state battery at different cathode areal capacities (0.5, 1, 2, 3, 4, and 5 mAh cm^-2^), C rates (0.001C, 0.2C, 0.5C, 1C, 2C, and 5C) and temperatures (30°C and 70°C). LLZO thickness is constant of 90 µm. LCO cathode is composed of 50 vol.% of LCO and 50 vol.% of LLZO.

**
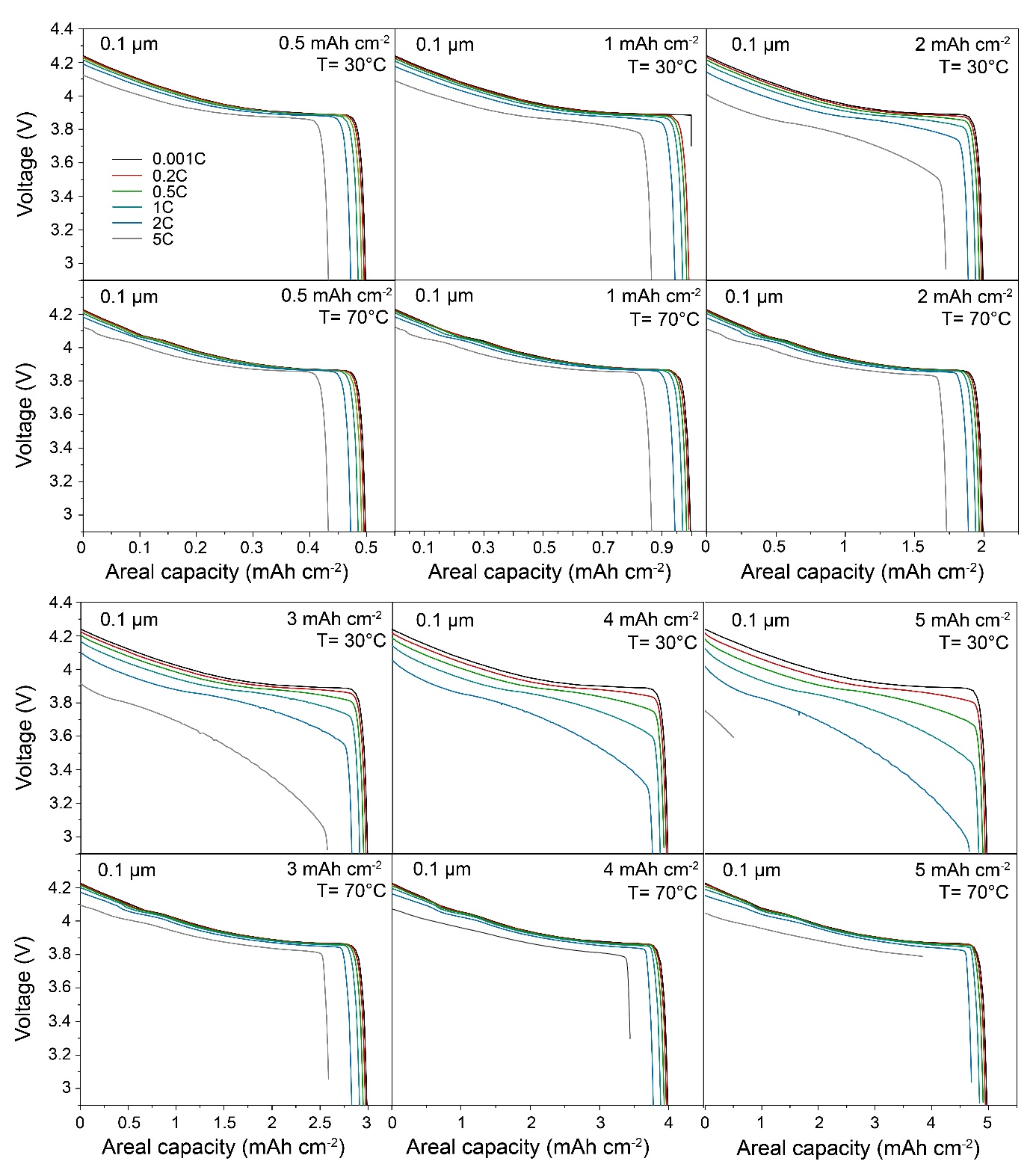
**

**Figures S20.** Simulated voltage profiles of Li/LLZO/LCO all-solid-state battery at different cathode areal capacities (0.5, 1, 2, 3, 4, and 5 mAh cm^-2^), C rates (0.001C, 0.2C, 0.5C, 1C, 2C, and 5C) and temperatures (30°C and 70°C). LLZO thickness is constant of 0.1 µm. LCO cathode is composed of 40 vol.% of LCO and 60 vol.% of LLZO.

**
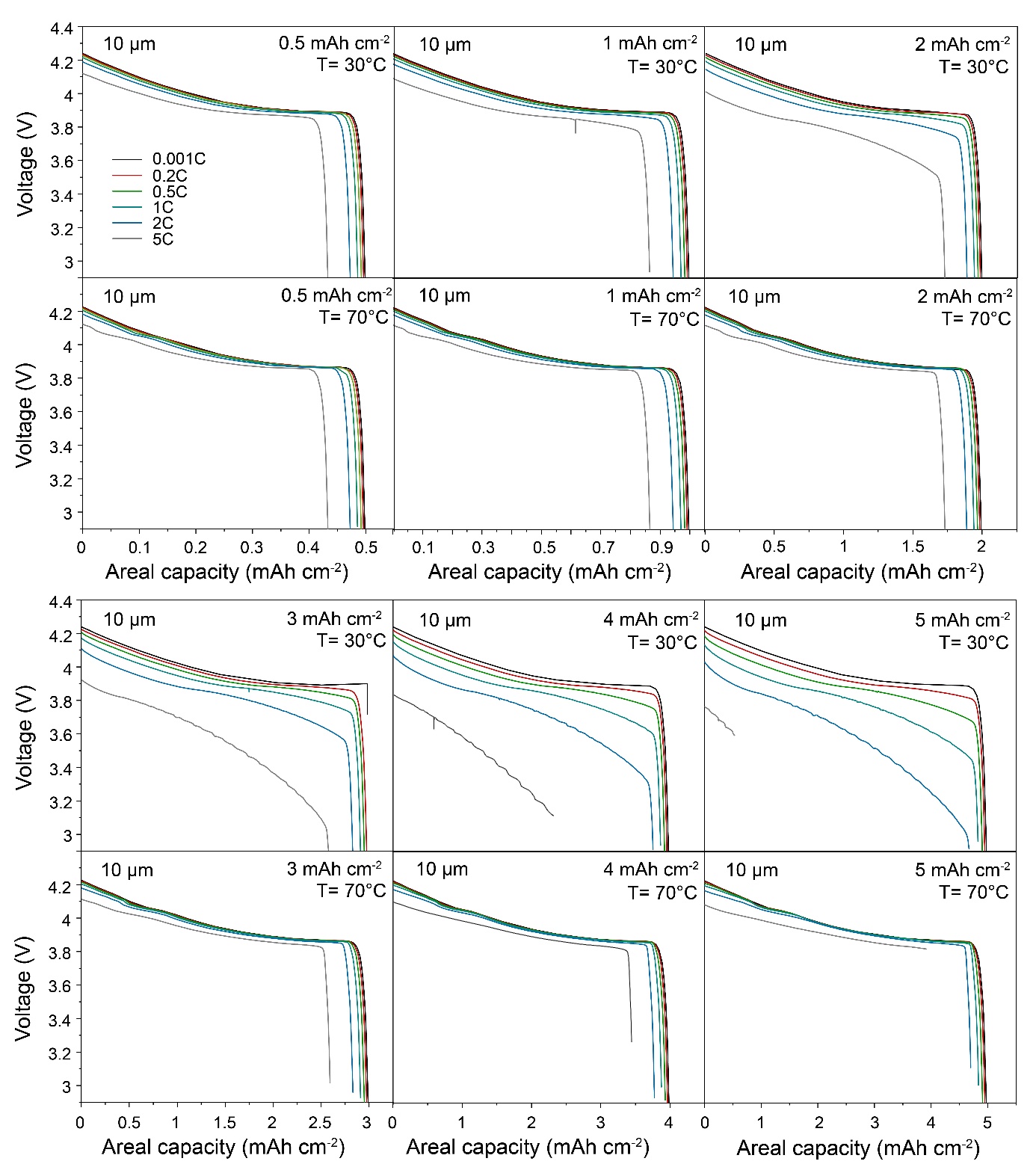
**

**Figures S21.** Simulated voltage profiles of Li/LLZO/LCO all-solid-state battery at different cathode areal capacities (0.5, 1, 2, 3, 4, and 5 mAh cm^-2^), C rates (0.001C, 0.2C, 0.5C, 1C, 2C, and 5C) and temperatures (30°C and 70°C). LLZO thickness is constant of 10 µm. LCO cathode is composed of 40 vol.% of LCO and 60 vol.% of LLZO.

**
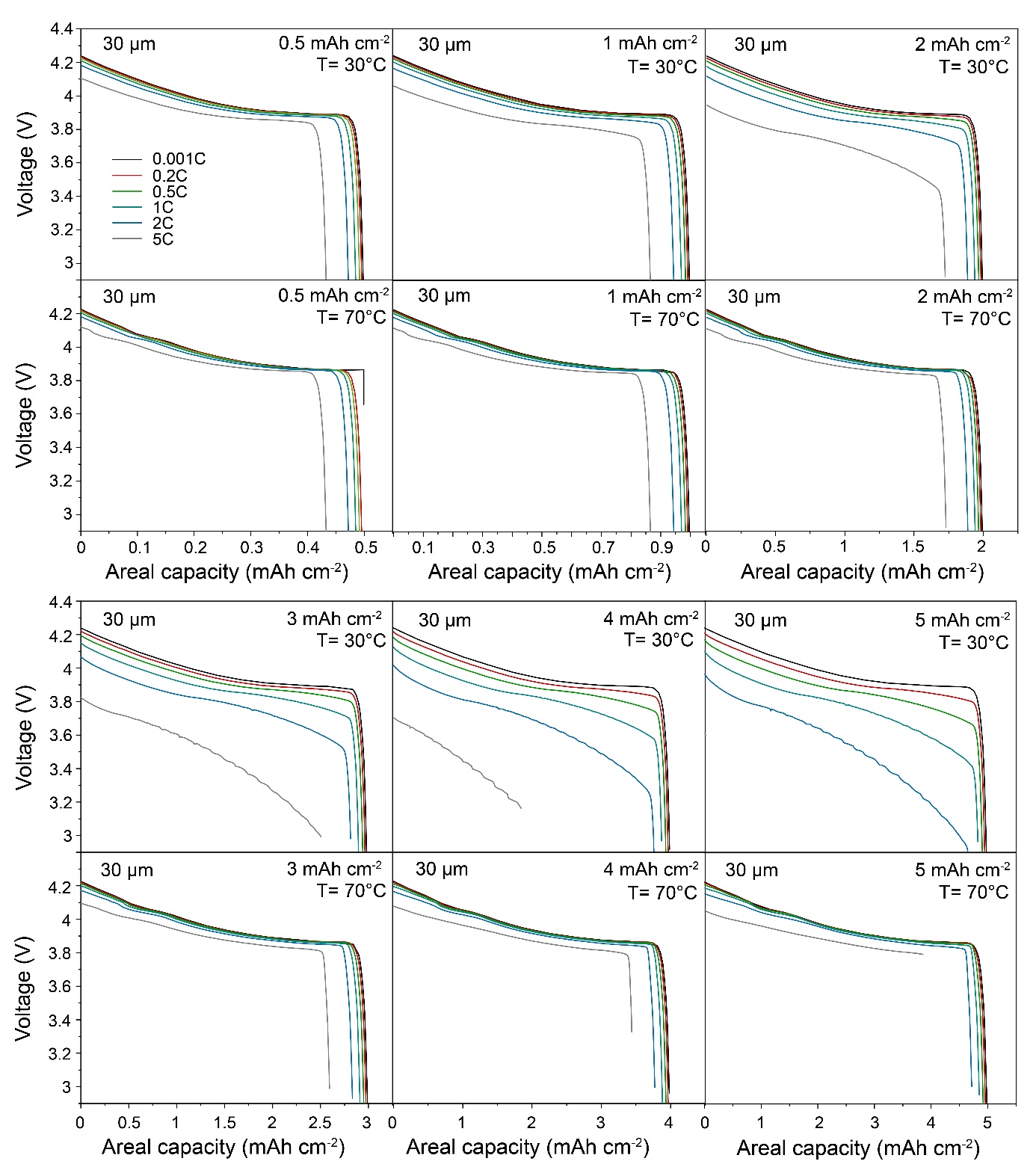
**

**Figures S22.** Simulated voltage profiles of Li/LLZO/LCO all-solid-state battery at different cathode areal capacities (0.5, 1, 2, 3, 4, and 5 mAh cm^-2^), C rates (0.001C, 0.2C, 0.5C, 1C, 2C, and 5C) and temperatures (30°C and 70°C). LLZO thickness is constant of 30 µm. LCO cathode is composed of 40 vol.% of LCO and 60 vol.% of LLZO.

**
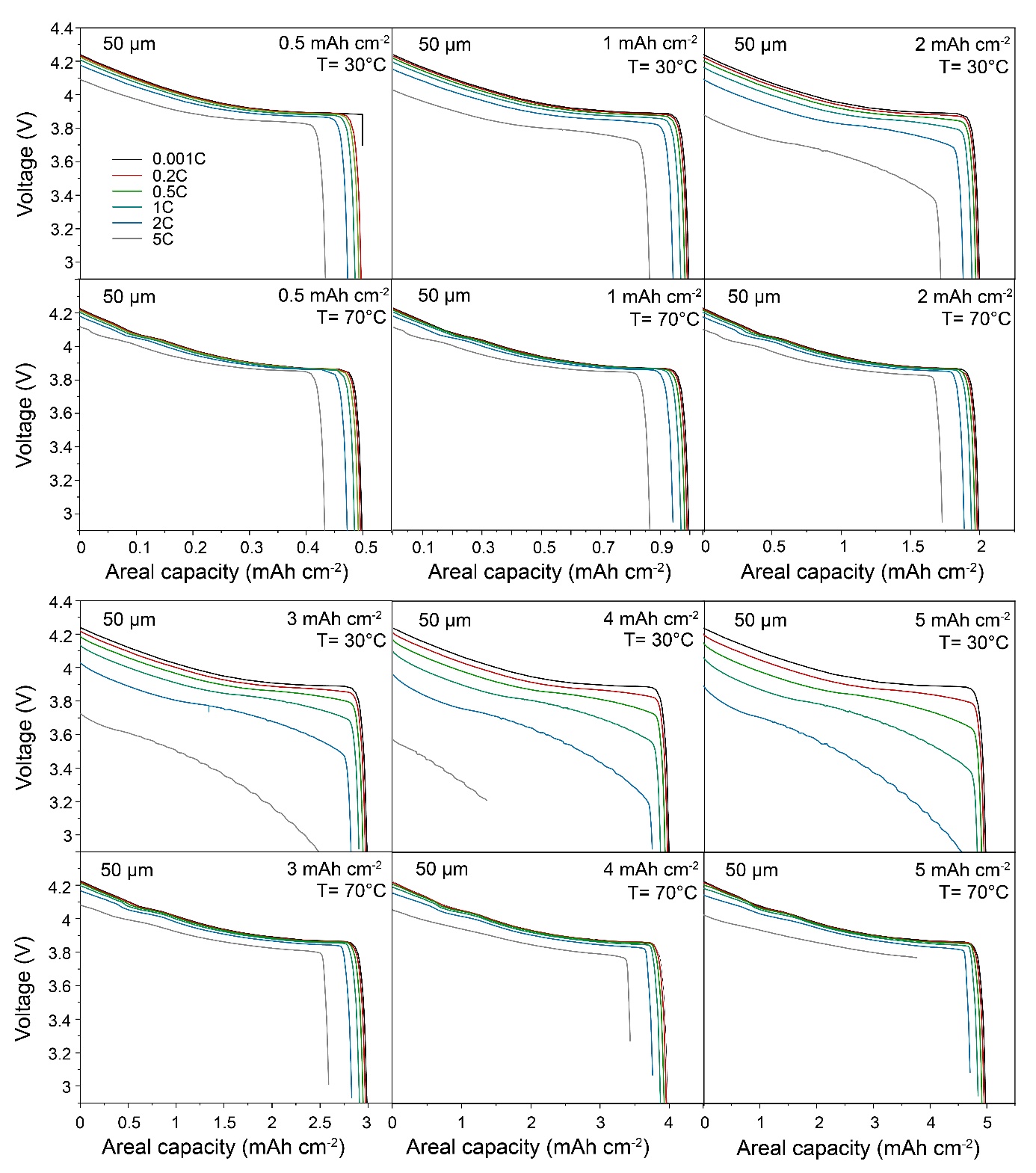
**

**Figures S23.** Simulated voltage profiles of Li/LLZO/LCO all-solid-state battery at different cathode areal capacities (0.5, 1, 2, 3, 4, and 5 mAh cm^-2^), C rates (0.001C, 0.2C, 0.5C, 1C, 2C, and 5C) and temperatures (30°C and 70°C). LLZO thickness is constant of 50 µm. LCO cathode is composed of 40 vol.% of LCO and 60 vol.% of LLZO.

**
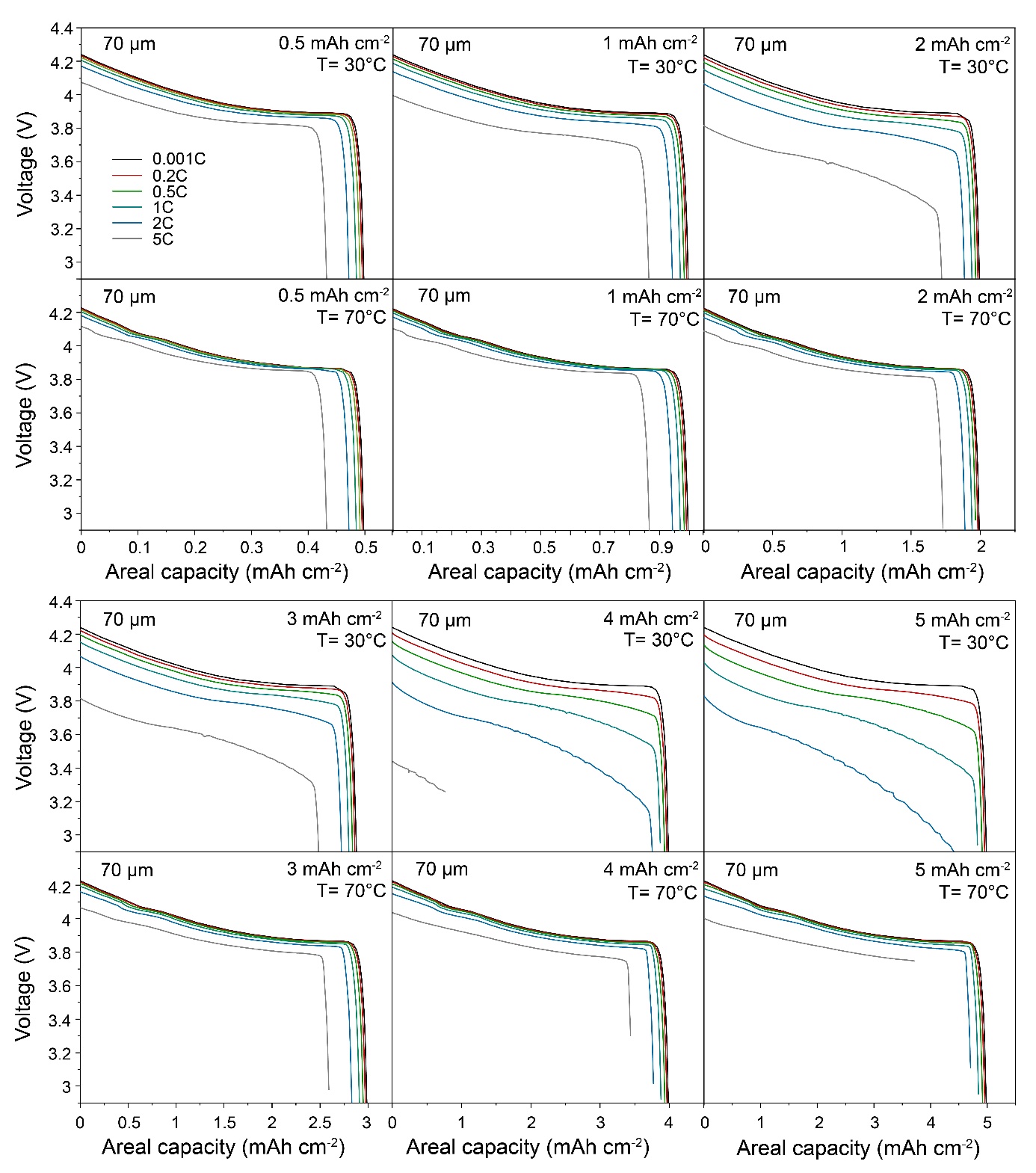
**

**Figures S24.** Simulated voltage profiles of Li/LLZO/LCO all-solid-state battery at different cathode areal capacities (0.5, 1, 2, 3, 4, and 5 mAh cm^-2^), C rates (0.001C, 0.2C, 0.5C, 1C, 2C, and 5C) and temperatures (30°C and 70°C). LLZO thickness is constant of 70 µm. LCO cathode is composed of 40 vol.% of LCO and 60 vol.% of LLZO.

**
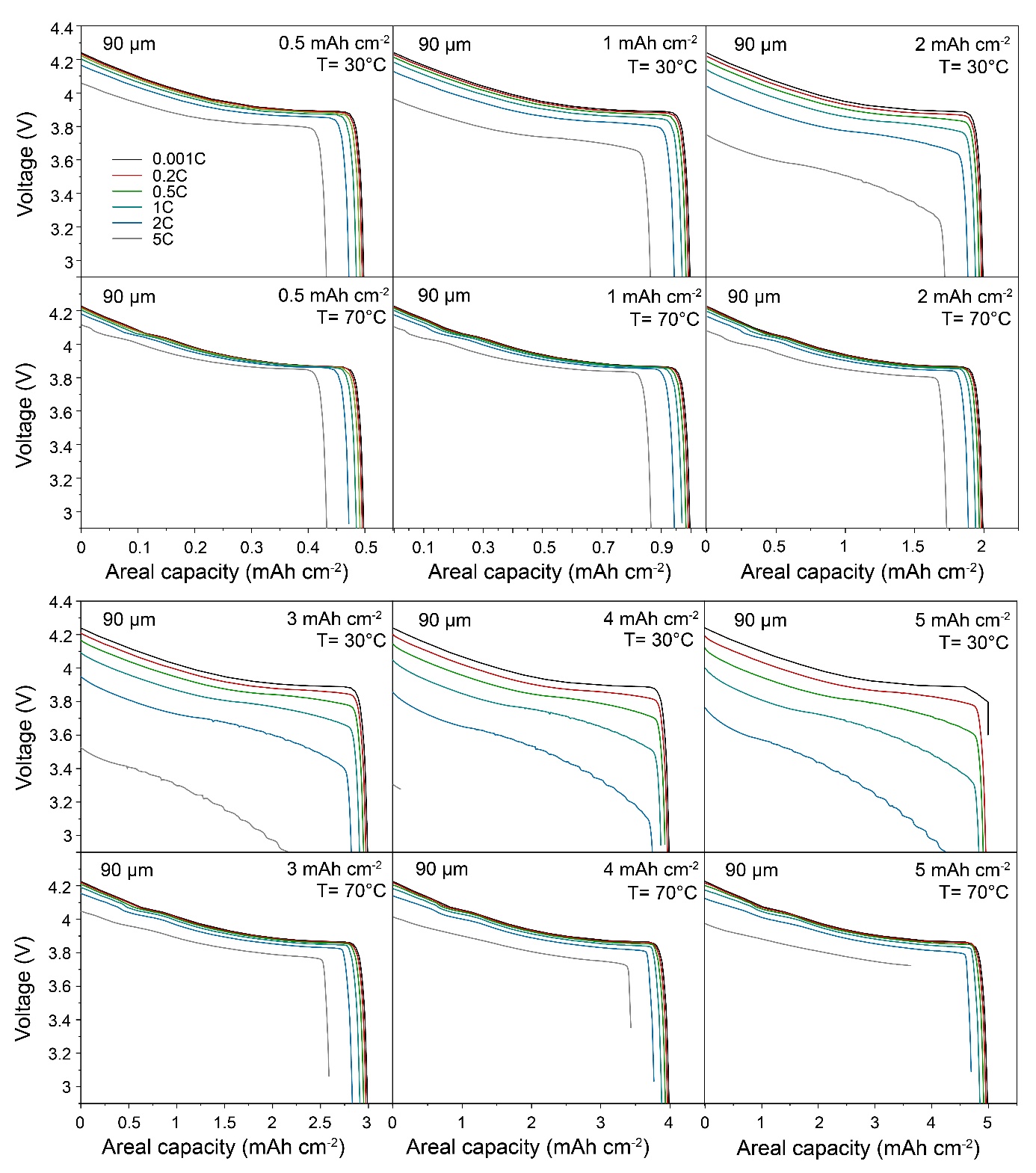
**

**Figures S25.** Simulated voltage profiles of Li/LLZO/LCO all-solid-state battery at different cathode areal capacities (0.5, 1, 2, 3, 4, and 5 mAh cm^-2^), C rates (0.001C, 0.2C, 0.5C, 1C, 2C, and 5C) and temperatures (30°C and 70°C). LLZO thickness is constant of 90 µm. LCO cathode is composed of 40 vol.% of LCO and 60 vol.% of LLZO.

**
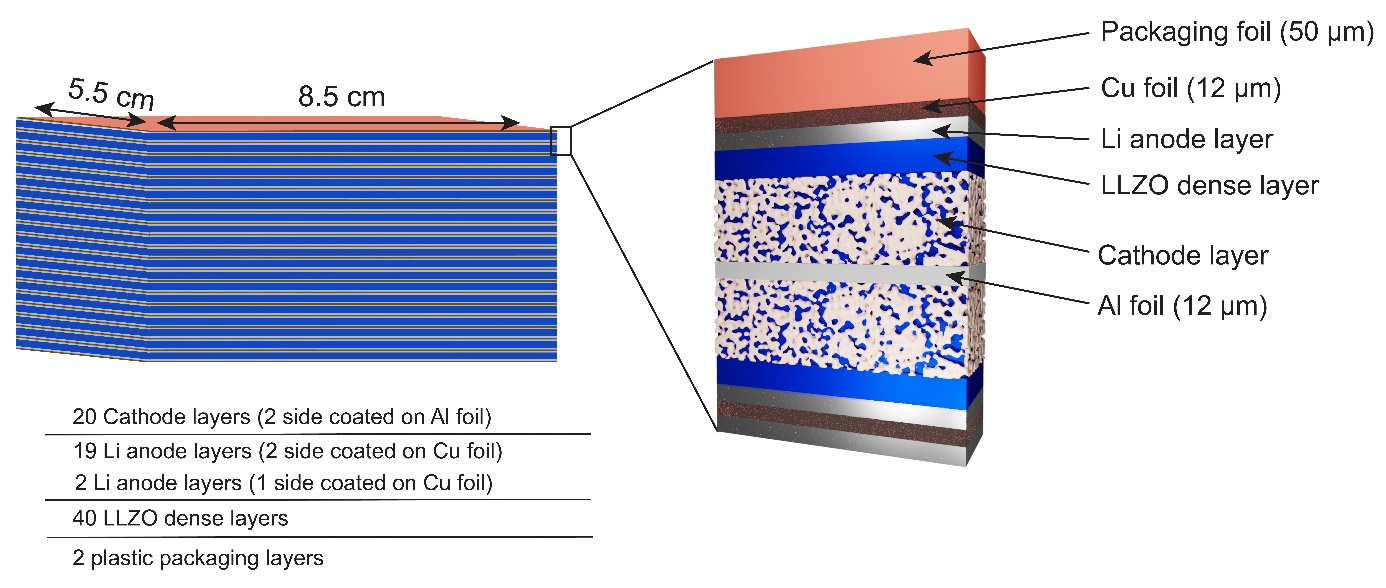
**

**Figure S26**. Schematics of the battery considered in this work for assessing the energy densities of Li-garnet SSBs.

**
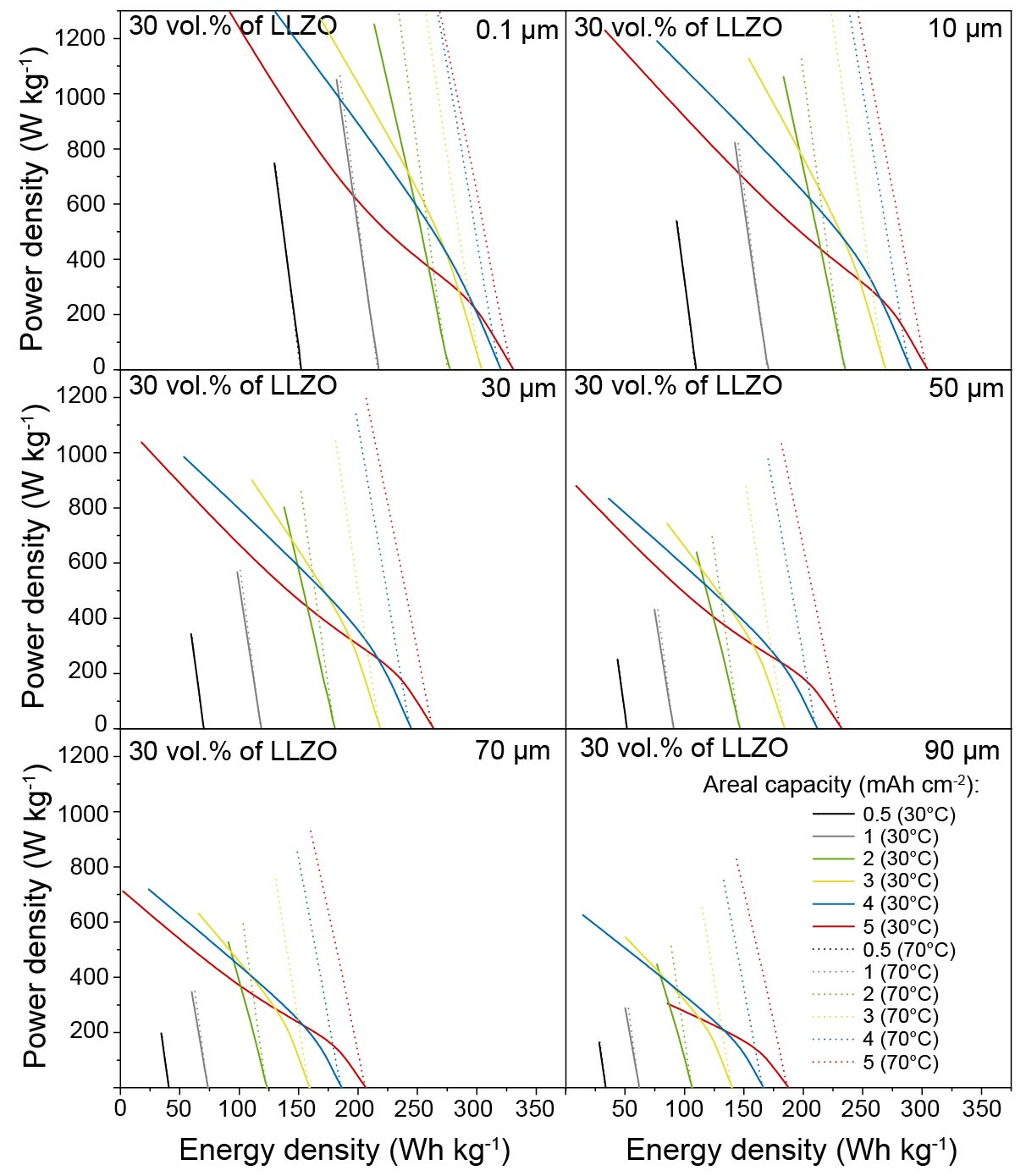
**

**Figures S27.** Gravimetric Ragone plots of Li/LLZO/LCO all-solid-state battery comprising LLZO solid electrolyte of different thicknesses (0.1 µm, 10 µm, 50 µm, 70 µm, and 90 µm) simulated at cathode areal capacities of 0.5, 1, 2, 3, 4, and 5 mAh cm^-2^, and temperatures of 30°C and 70°C. The composition of LCO cathode is constant (70 vol.% of LCO and 30 vol.% of LLZO).

**
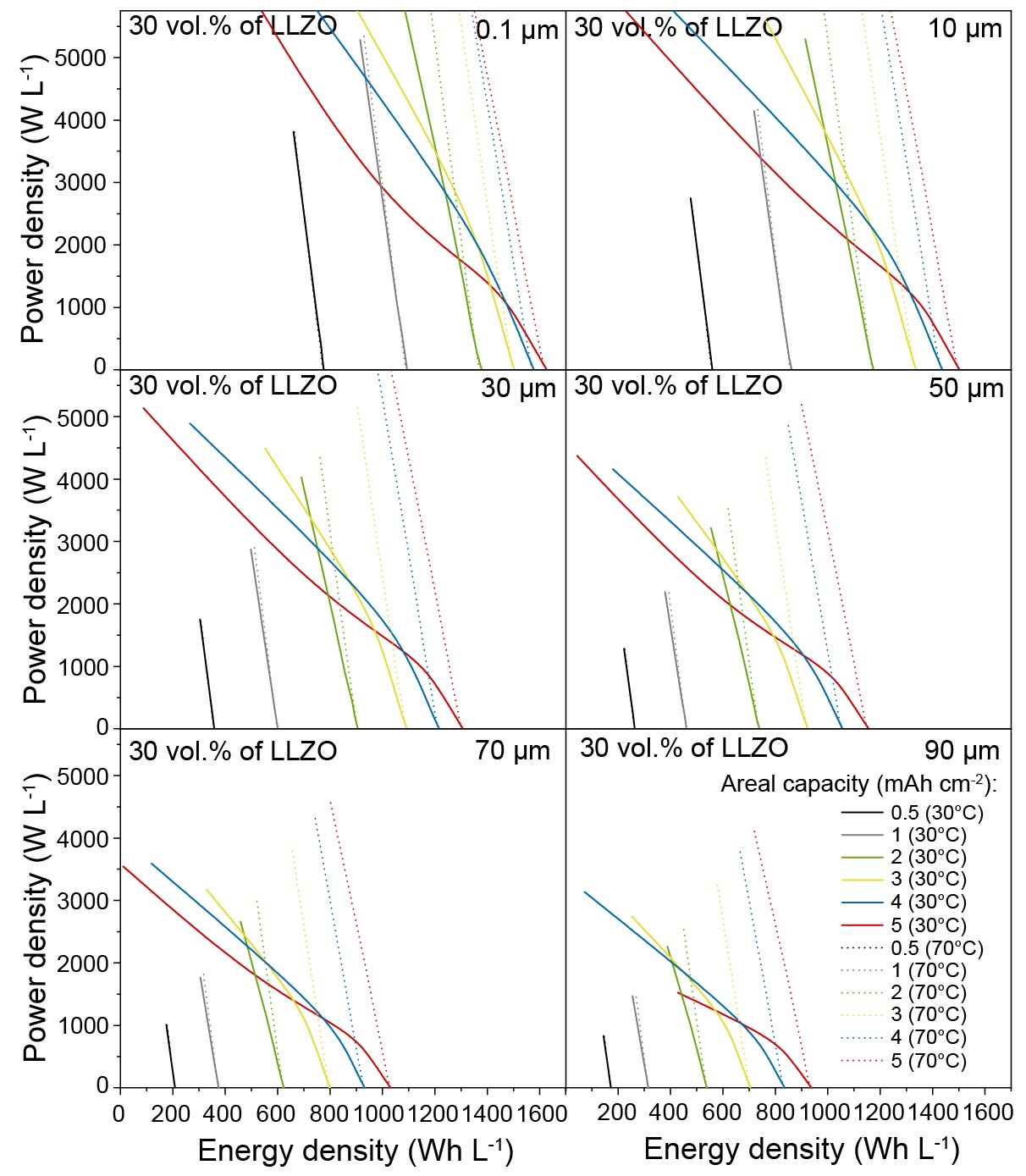
**

**Figures S28.** Volumetric Ragone plots of Li/LLZO/LCO all-solid-state battery comprising LLZO solid electrolyte of different thicknesses (0.1 µm, 10 µm, 50 µm, 70 µm, and 90 µm) simulated at cathode areal capacities of 0.5, 1, 2, 3, 4, and 5 mAh cm^-2^, and temperatures of 30°C and 70°C. The composition of LCO cathode is constant (70 vol.% of LCO and 30 vol.% of LLZO).


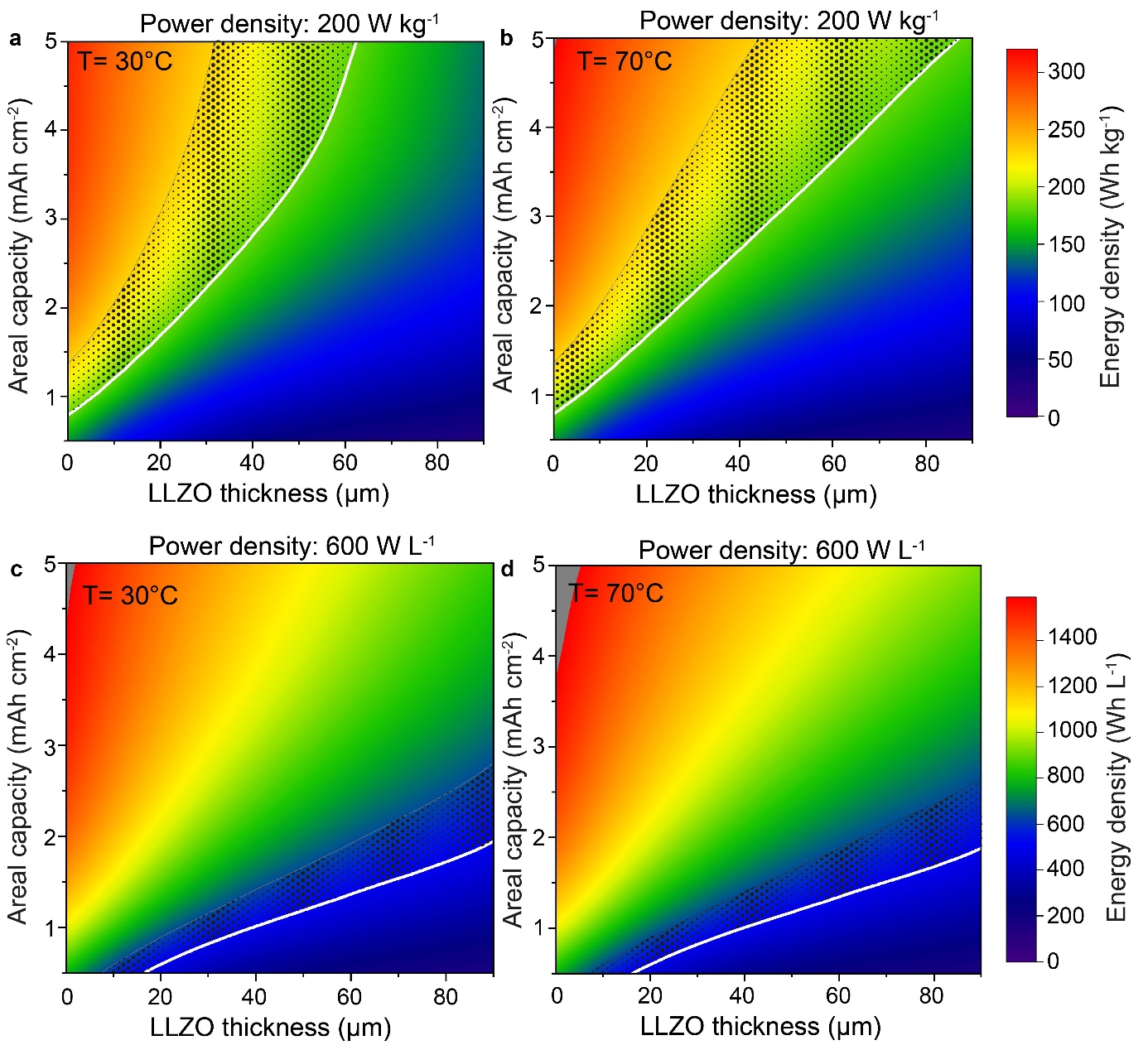


**Figure S29.** Simulated gravimetric (a, b) and volumetric (c, d) energy densities of Li-free/LLZO/LCO all-solid-state battery vs. cathode areal capacity and LLZO thickness plotted at power densities of 200 W kg^-1^ and 600 W L^-1^ and temperatures of 30°C and 70°C. The composition of LCO cathode is constant (70 vol.% of LCO and 30 vol.% of LLZO).

**
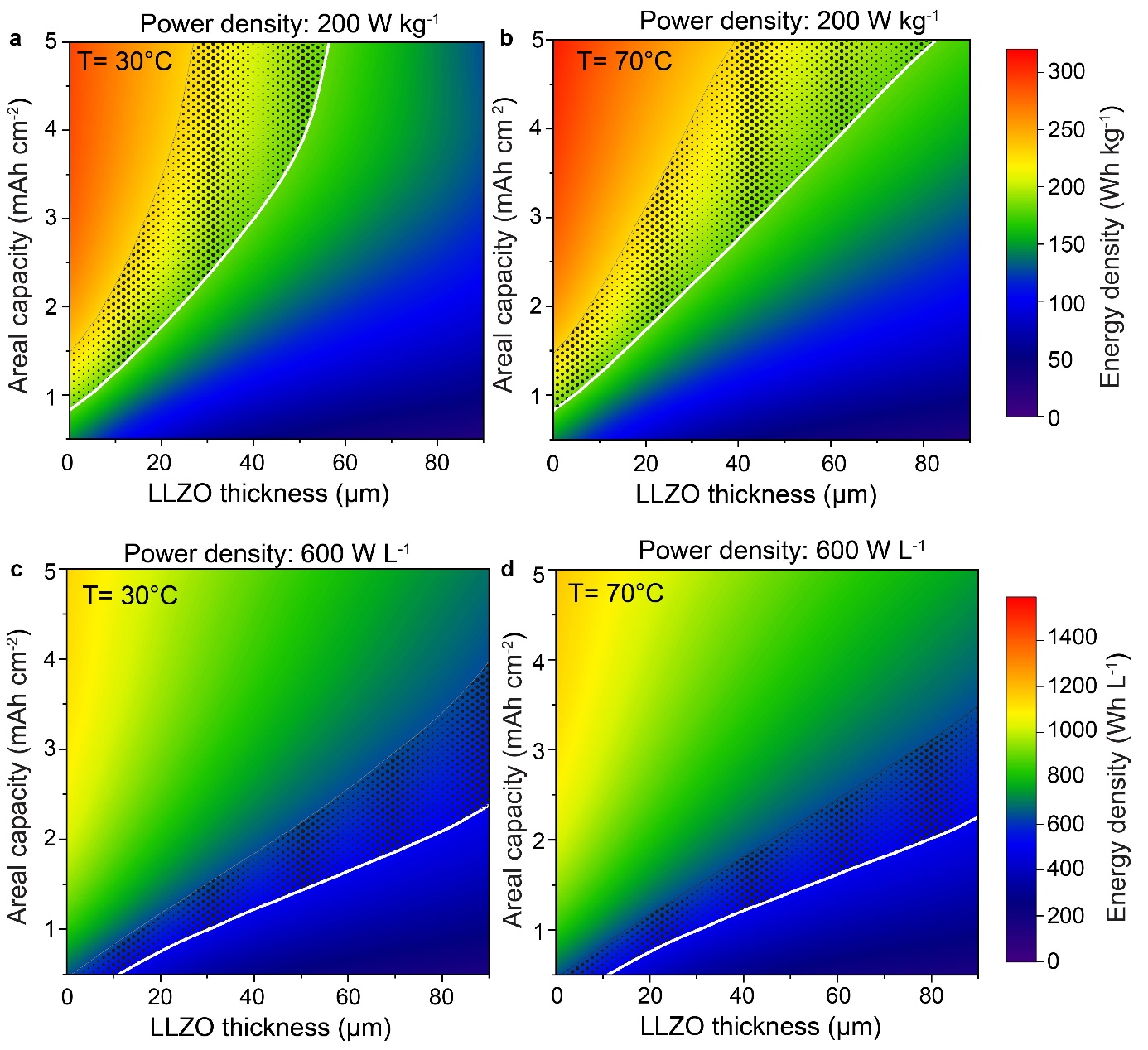
**

**Figure S30.** Simulated gravimetric (a, b) and volumetric (c, d) energy densities of Li/LLZO/LCO all-solid-state battery vs. cathode areal capacity and LLZO thickness plotted at power densities of 200 W kg^-1^ and 600 W L^-1^ and temperatures of 30°C and 70°C. The composition of LCO cathode is constant (70 vol.% of LCO and 30 vol.% of LLZO). The areal capacity (thickness) of the Li metal anode corresponds to 200% of cathode areal capacity.

**
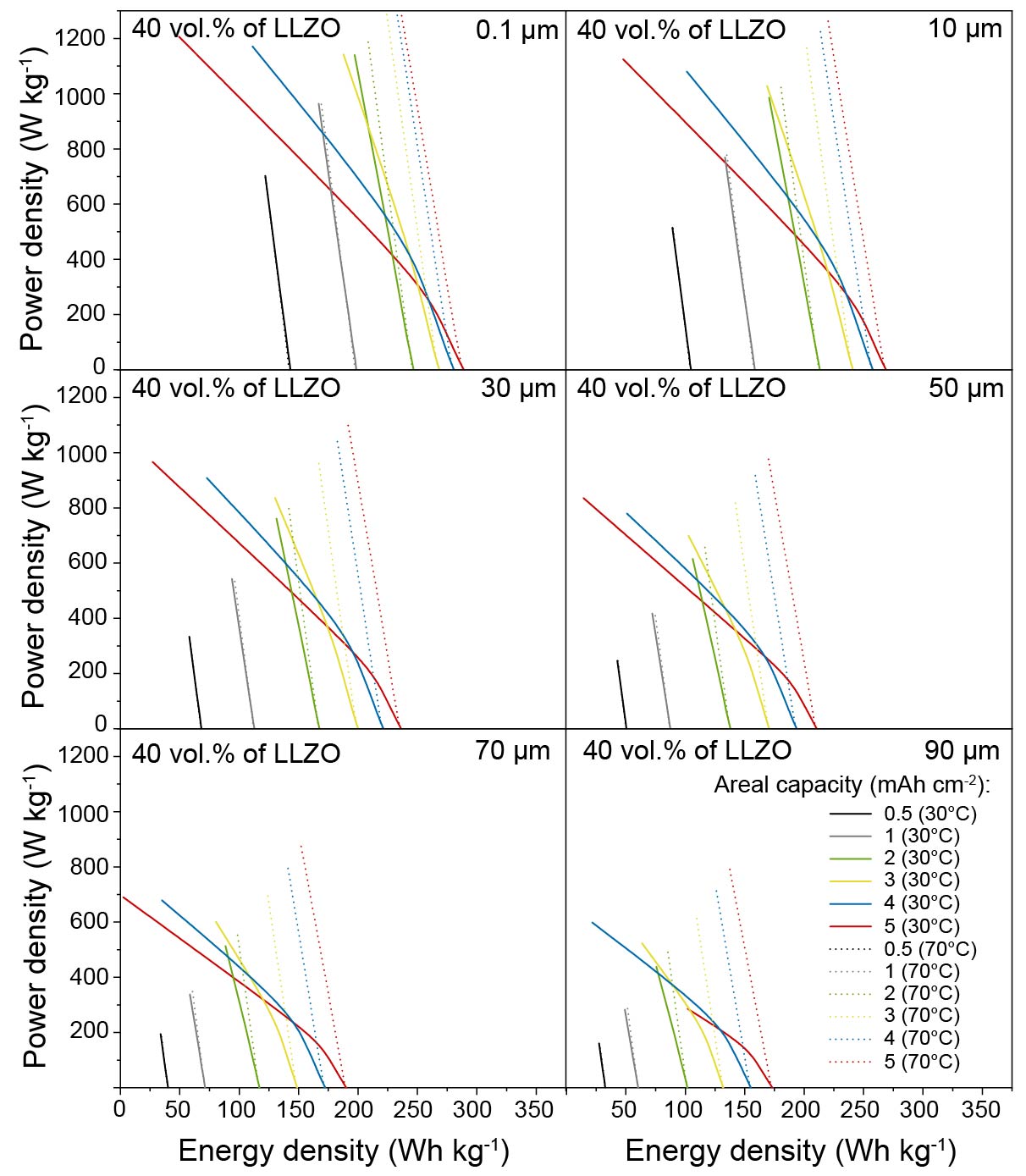
**

**Figures S31.** Gravimetric Ragone plots of Li/LLZO/LCO all-solid-state battery comprising LLZO solid electrolyte of different thicknesses (0.1 µm, 10 µm, 50 µm, 70 µm, and 90 µm) simulated at cathode areal capacities of 0.5, 1, 2, 3, 4, and 5 mAh cm^-2^, and temperatures of 30°C and 70°C. The composition of LCO cathode is constant (60 vol.% of LCO and 40 vol.% of LLZO).

**
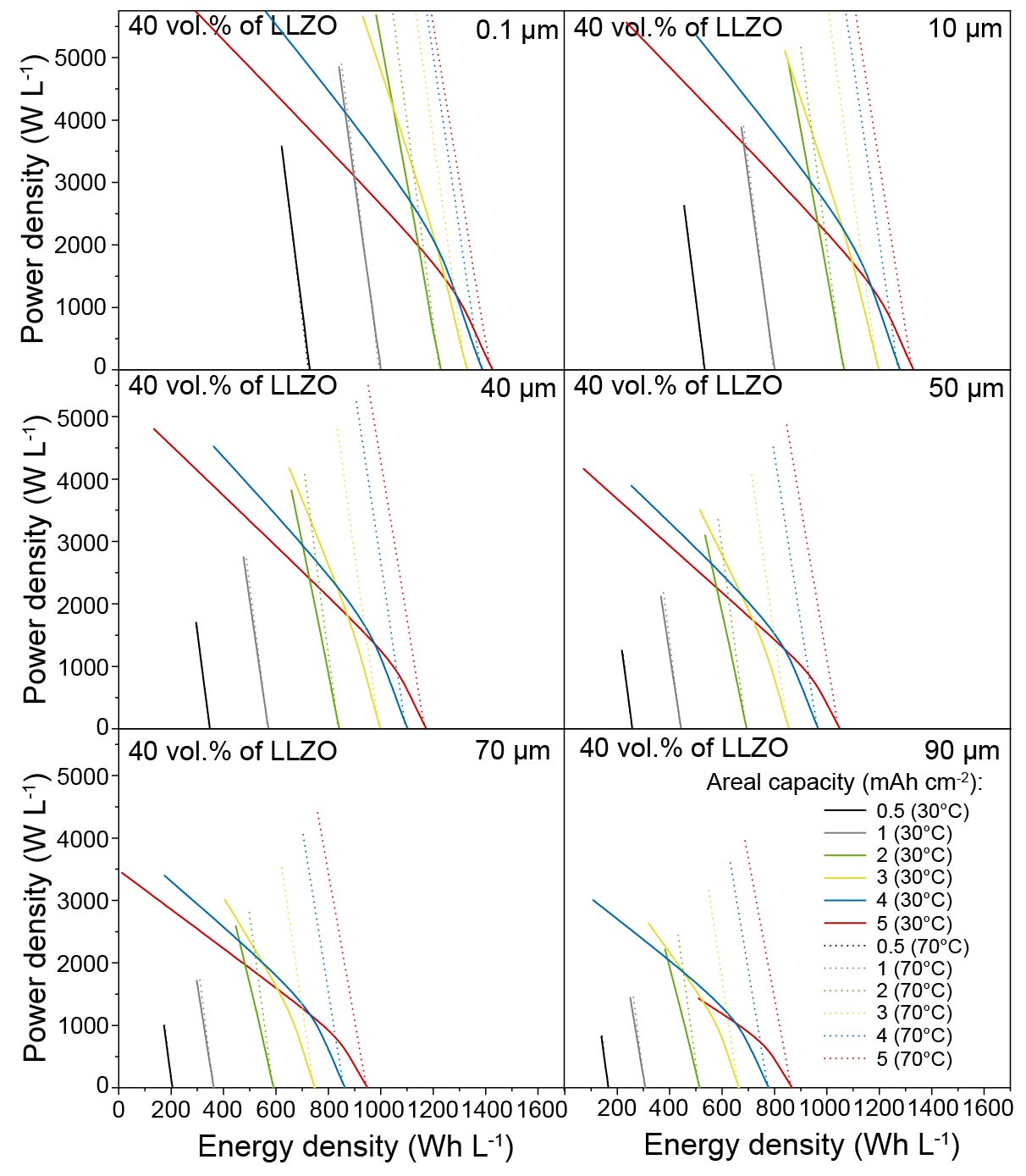
**

**Figures S32.** Volumetric Ragone plots of Li/LLZO/LCO all-solid-state battery comprising LLZO solid electrolyte of different thicknesses (0.1 µm, 10 µm, 50 µm, 70 µm, and 90 µm) simulated at cathode areal capacities of 0.5, 1, 2, 3, 4, and 5 mAh cm^-2^, and temperatures of 30°C and 70°C. The composition of LCO cathode is constant (60 vol.% of LCO and 40 vol.% of LLZO).


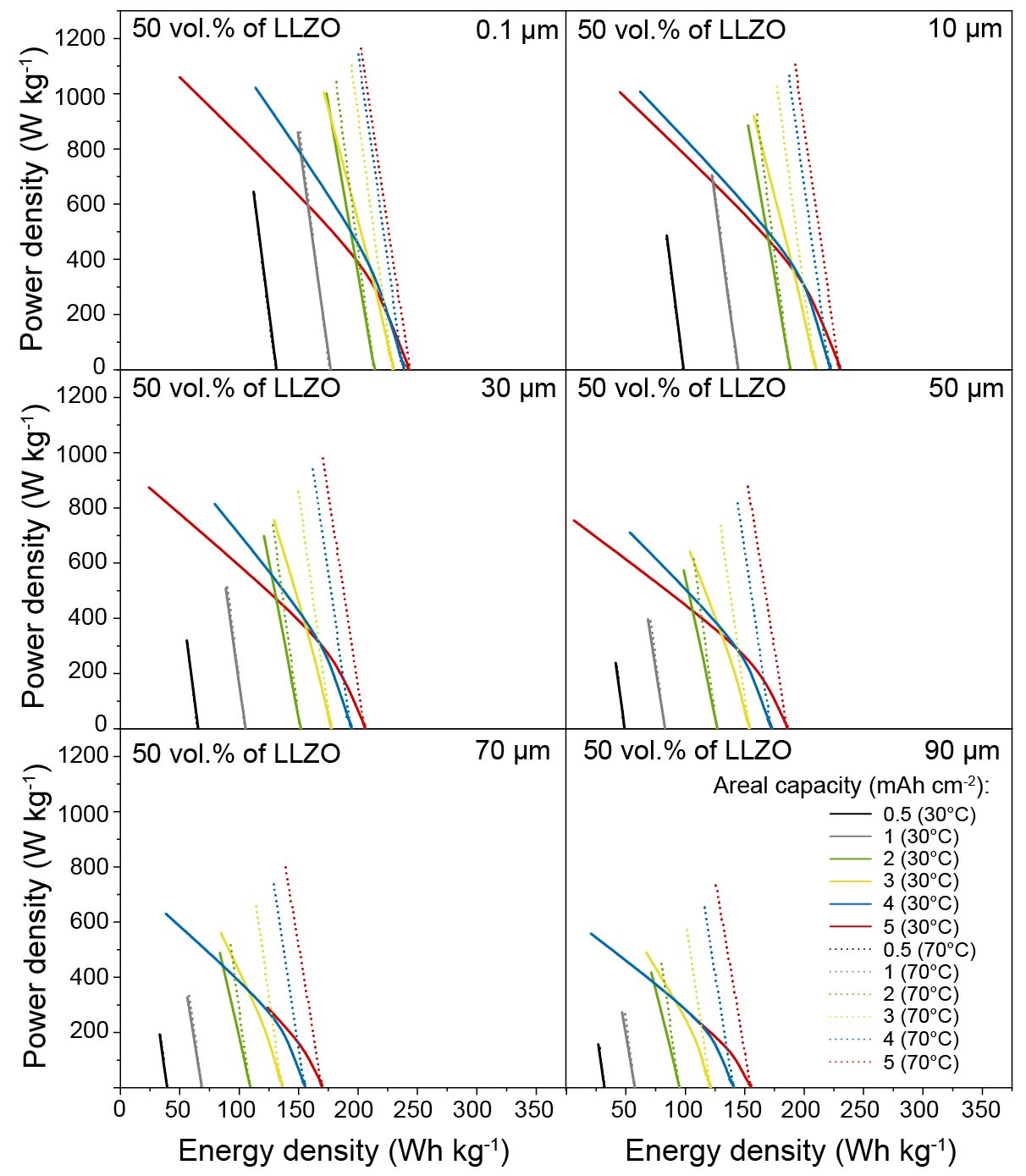


**Figures S33.** Gravimetric Ragone plots of Li/LLZO/LCO all-solid-state battery comprising LLZO solid electrolyte of different thicknesses (0.1 µm, 10 µm, 50 µm, 70 µm, and 90 µm) simulated at cathode areal capacities of 0.5, 1, 2, 3, 4, and 5 mAh cm^-2^, and temperatures of 30°C and 70°C. The composition of LCO cathode is constant (50 vol.% of LCO and 50 vol.% of LLZO).

**
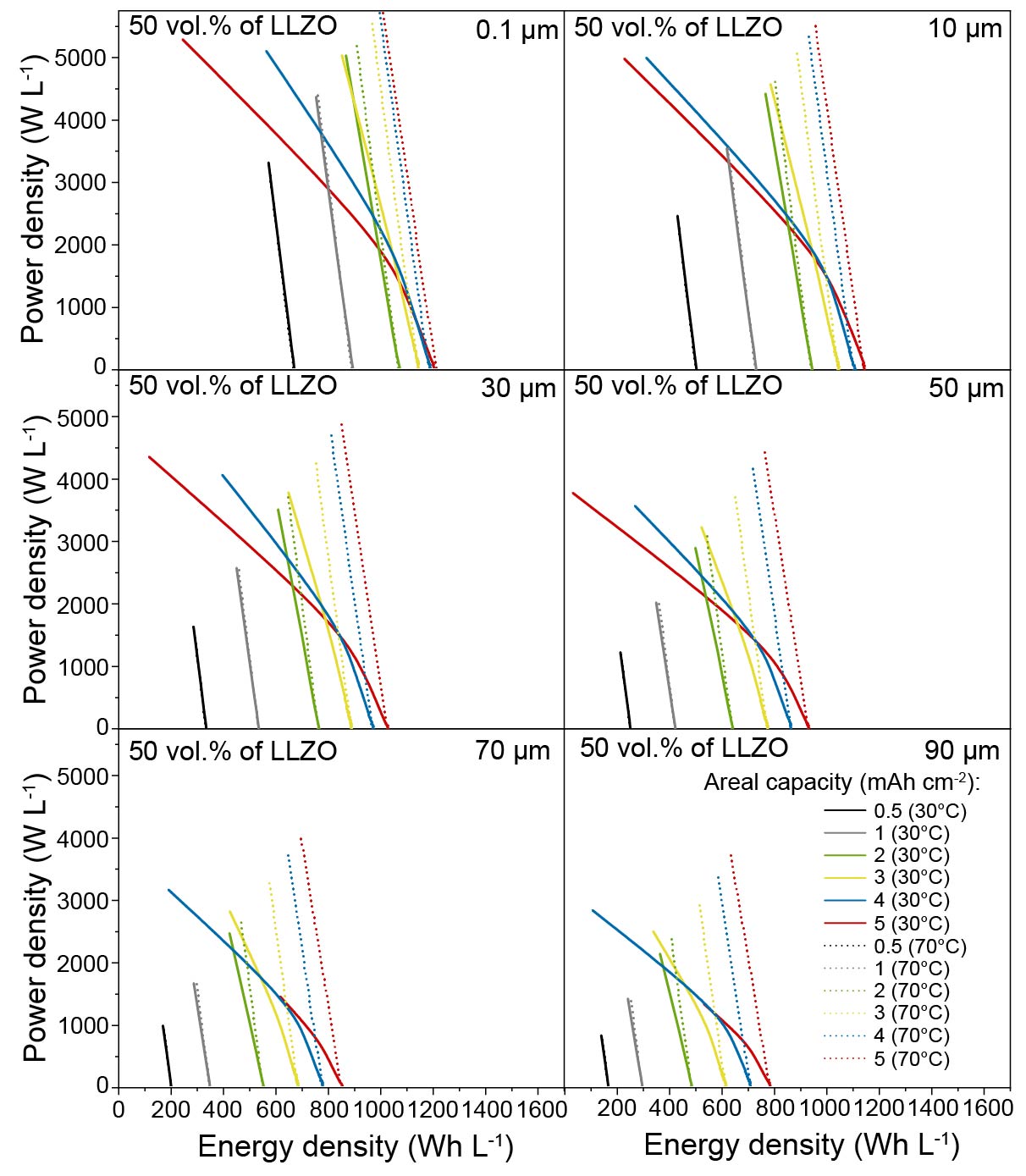
**

**Figures S34.** Volumetric Ragone plots of Li/LLZO/LCO all-solid-state battery comprising LLZO solid electrolyte of different thicknesses (0.1 µm, 10 µm, 50 µm, 70 µm, and 90 µm) simulated at cathode areal capacities of 0.5, 1, 2, 3, 4, and 5 mAh cm^-2^, and temperatures of 30°C and 70°C. The composition of LCO cathode is constant (50 vol.% of LCO and 50 vol.% of LLZO).

**
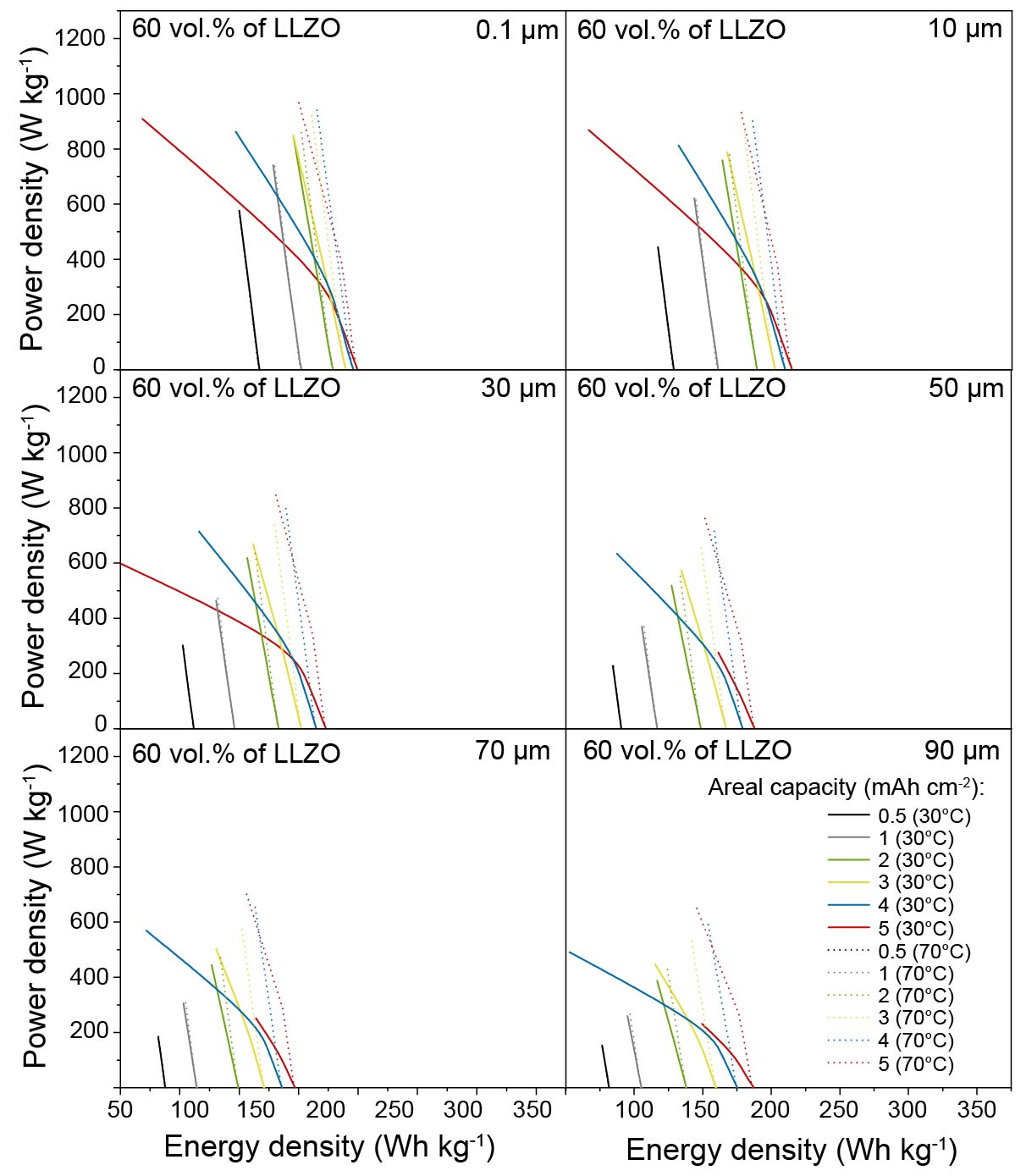
**

**Figures S35.** Gravimetric Ragone plots of Li/LLZO/LCO all-solid-state battery comprising LLZO solid electrolyte of different thicknesses (0.1 µm, 10 µm, 50 µm, 70 µm, and 90 µm) simulated at cathode areal capacities of 0.5, 1, 2, 3, 4, and 5 mAh cm^-2^, and temperatures of 30°C and 70°C. The composition of LCO cathode is constant (40 vol.% of LCO and 60 vol.% of LLZO).

**
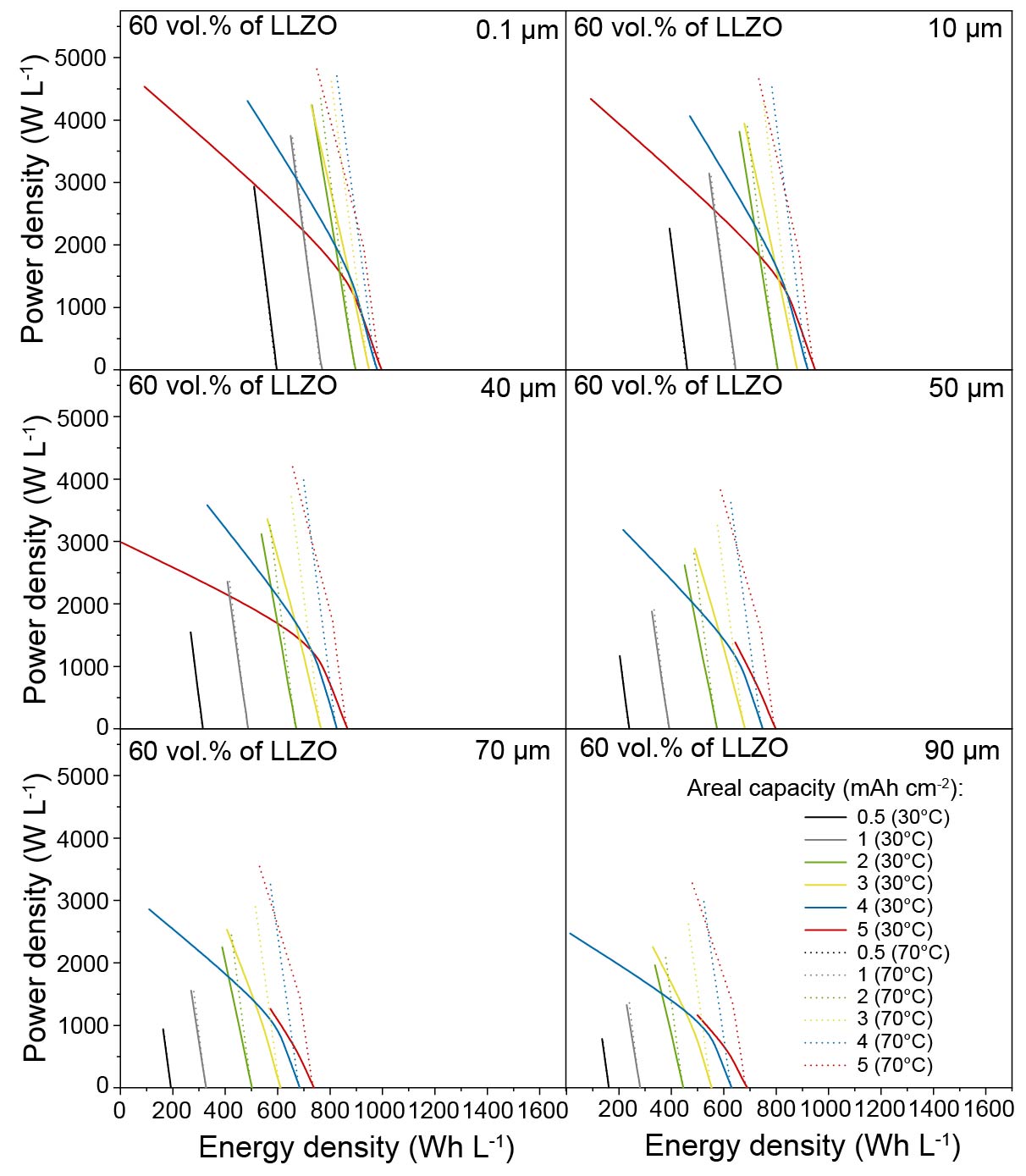
**

**Figures S36.** Volumetric Ragone plots of Li/LLZO/LCO all-solid-state battery comprising LLZO solid electrolyte of different thicknesses (0.1 µm, 10 µm, 50 µm, 70 µm, and 90 µm) simulated at cathode areal capacities of 0.5, 1, 2, 3, 4, and 5 mAh cm^-2^, and temperatures of 30°C and 70°C. The composition of LCO cathode is constant (40 vol.% of LCO and 60 vol.% of LLZO).

**
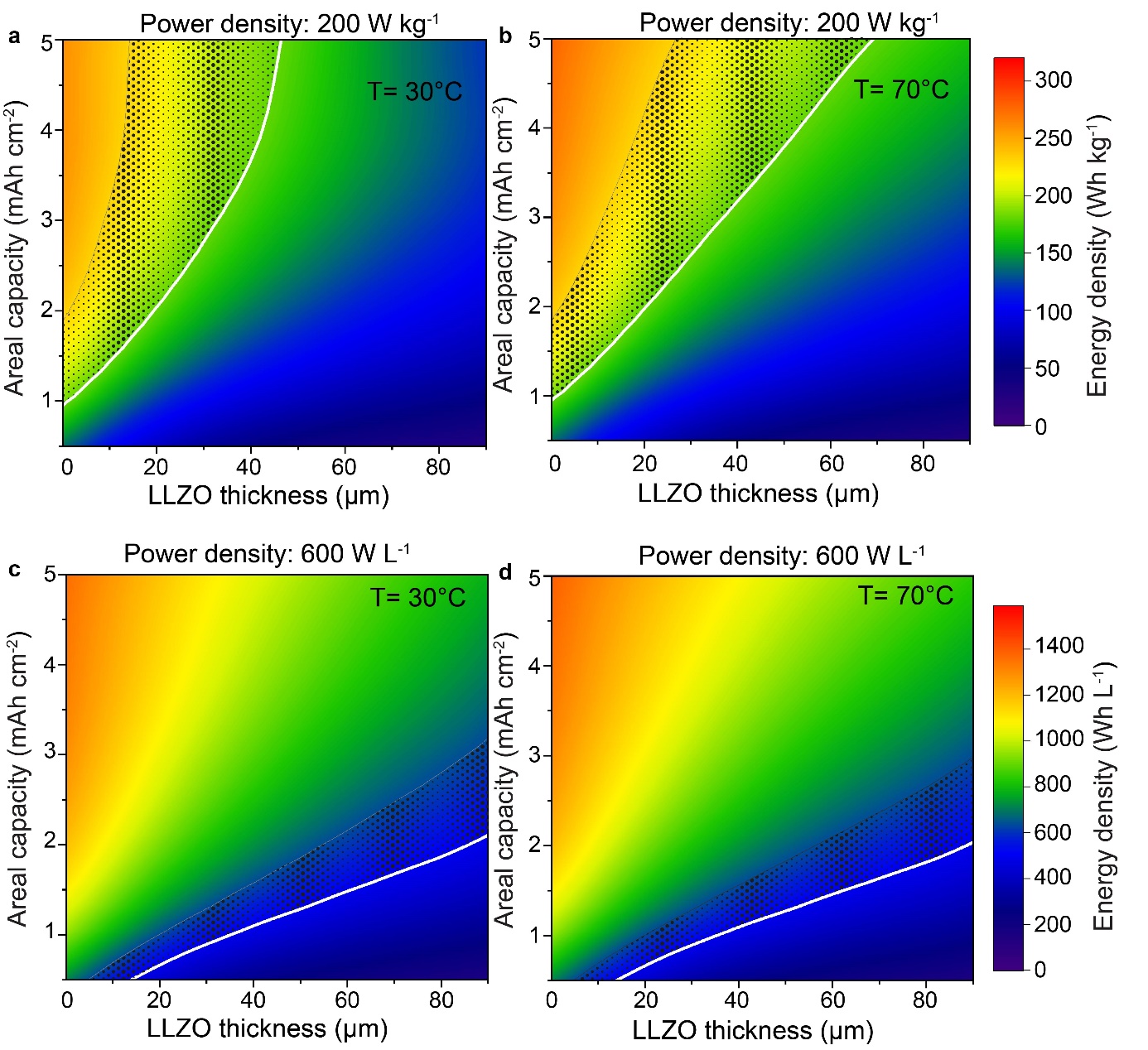
**

**Figure S37.** Simulated gravimetric (a, b) and volumetric (c, d) energy densities of Li/LLZO/LCO all-solid-state battery vs. cathode areal capacity and LLZO thickness plotted at power densities of 200 W kg^-1^ and 600 W L^-1^ and temperatures of 30°C and 70°C. The composition of LCO cathode is constant (60 vol.% of LCO and 40 vol.% of LLZO). The areal capacity (thickness) of the Li metal anode corresponds to 20% of cathode areal capacity.

**
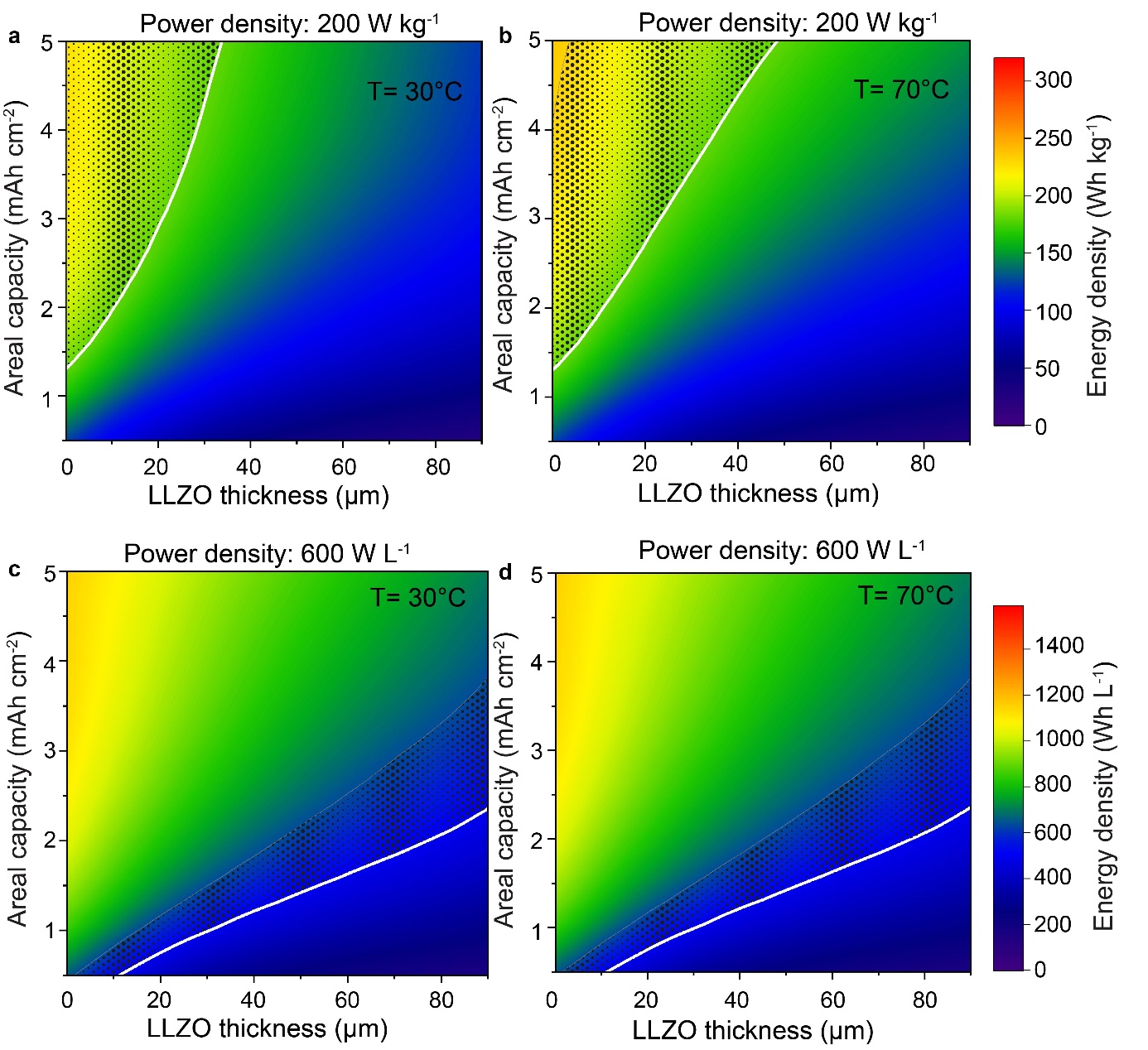
**

**Figure S38.** Simulated gravimetric (a, b) and volumetric (c, d) energy densities of Li/LLZO/LCO all-solid-state battery vs. cathode areal capacity and LLZO thickness plotted at power densities of 200 W kg^-1^ and 600 W L^-1^ and temperatures of 30°C and 70°C. The composition of LCO cathode is constant (50 vol.% of LCO and 50 vol.% of LLZO). The areal capacity (thickness) of the Li metal anode corresponds to 20% of cathode areal capacity.

**
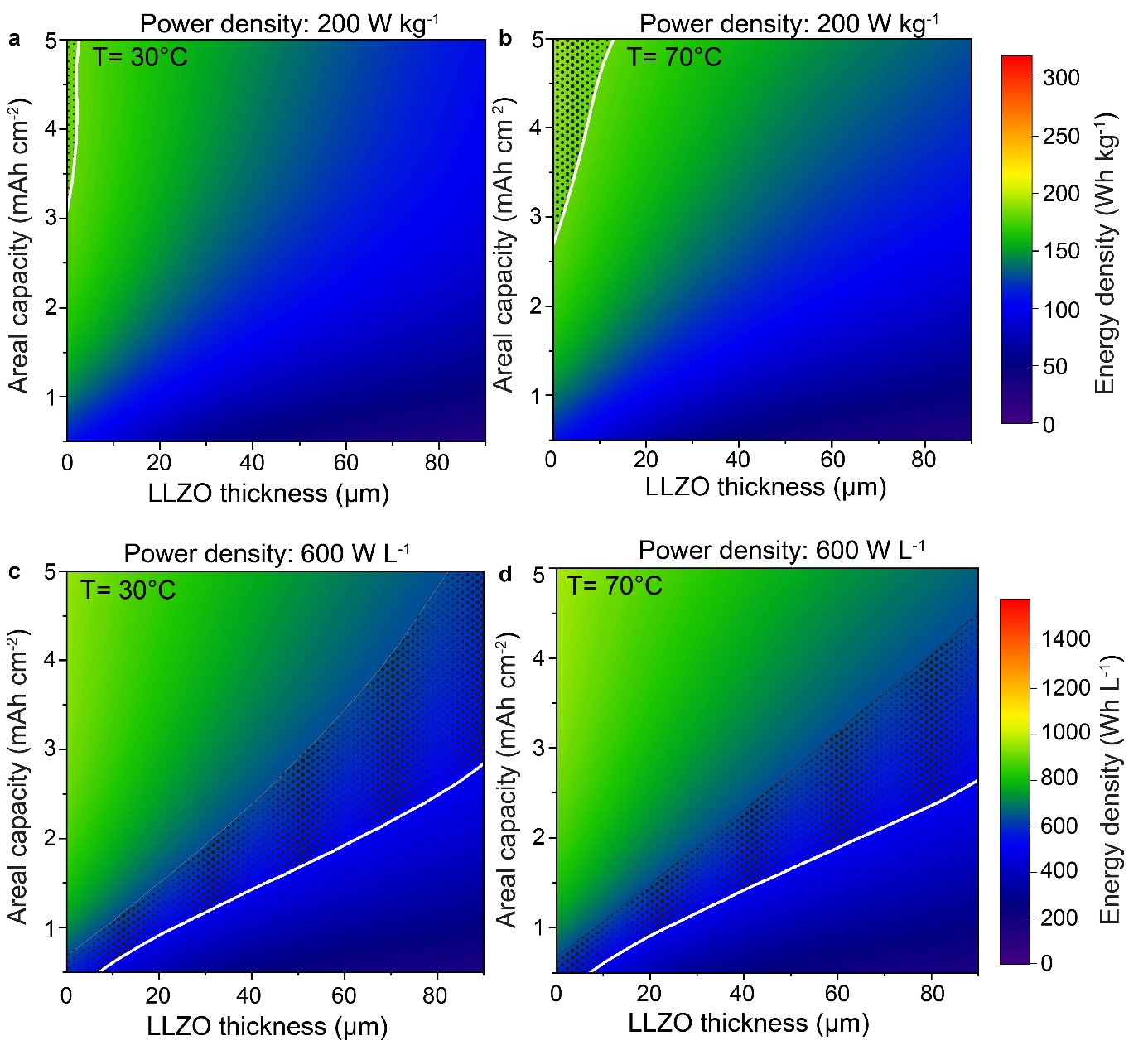
**

**Figure S39.** Simulated gravimetric (a, b) and volumetric (c, d) energy densities of Li/LLZO/LCO all-solid-state battery vs. cathode areal capacity and LLZO thickness plotted at power densities of 200 W kg^-1^ and 600 W L^-1^ and temperatures of 30°C and 70°C. The composition of LCO cathode is constant (40 vol.% of LCO and 60 vol.% of LLZO). The areal capacity (thickness) of the Li metal anode corresponds to 20% of cathode areal capacity.

**
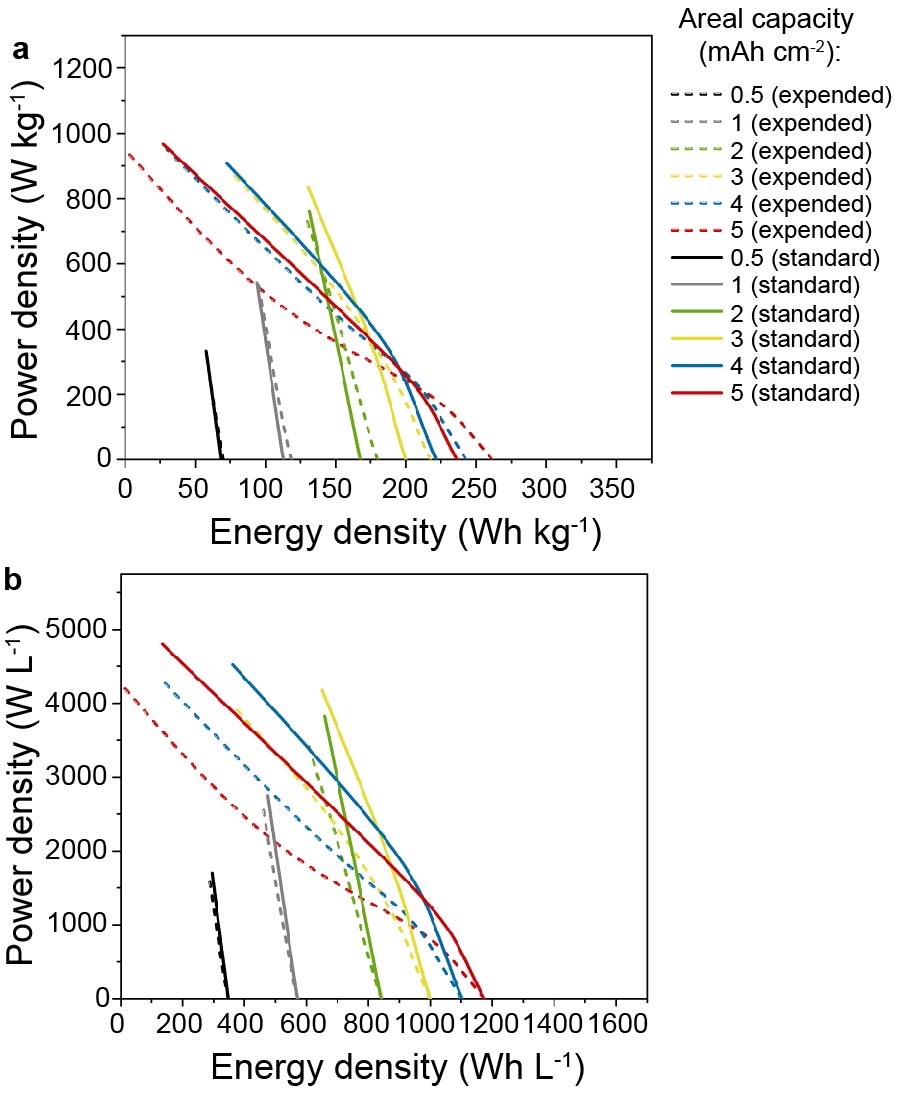
**

**Figure S40.** Comparison of gravimetric (a) and volumetric (b) Ragone plots of the solid-state system (Li/LLZO/LLZO+LCO) obtained using standard and expended simulations (T = 30°C). Expended simulations were performed by integrating additional parameters, such as LLZO/Li (0.1 Ω cm^2^) and LLZO/LCO (50 Ω cm^2^) interfacial resistances and the 13% porosity in LCO/LLZO solid-state cathode. Cathode composition for standard simulation was 60 vol.% of LCO/40 vol.% of LLZO. Cathode composition for expended simulations was 60 vol.% of LCO/27 vol.% of LLZO/13 vol.% of porosity.

**Table S1.** Parameters used for simulations.

| Parameters | Value | Symbol |
| --- | --- | --- |
| Ionic conductivity of LLZO electrolyte at 30°C | 2.85·10^-4^ S m^-1^ | $\sigma_{LLZO}$ |
| Ionic conductivity of LLZO electrolyte at 70°C | 1.7·10^-3^ S m^-1^ | $\sigma_{LLZO}$ |
| Anodic apparent transfer coefficient | 0.5 | $\alpha_{a}$ |
| Cathodic apparent transfer coefficient | 0.5 | $\alpha_{c}$ |
| Reference exchange current density of negative metallic lithium electrode | 400 A m^-2^ | $i_{0,ref}$ |
| Reference exchange current density of positive LCO electrode | 0.0543 A m^-2^ | $i_{0,ref}$ |
| Initial concentration of Li ions in Positive Electrode | 44949 mol m^-3^ | $c_{{Li}^{+},init}$ |
| Maximum concentration of Li ions in positive Electrode | 49973 mol m^-3^ | $c_{{Li}^{+},max}$ |
| Concentration of Li per volume of negative electrode | 77111 mol m^-3^ | $c_{{Li}^{+},max}$ |
| Minimum state of charge of positive electrode | 0.43 | ${soc}_{min}$ |
| Maximum electrode state of charge of positive Electrode | 0.93 | ${soc}_{max}$ |
| Electronic conductivity of positive electrode | 0.113 S m^-1^ | $\sigma_{LCO}$ |
| Electronic conductivity of negative electrode | 6.0·10^5^ S m^-1^ | $\sigma$ |
| Diffusivity of positive electrode | 5.0·10^-13^ m^2^ s^-1^ | $D_{{Li}^{+}}$ |
| Particle radius in positive electrode | 5.0·10^-7^ m | $r_{p}$ |

**Table S2.** Parameters used for the calculations of energy density.

| Parameters | Value |
| --- | --- |
| Battery dimensions | 5.5 cm _˟_ 8.5 cm |
| Density of Li | 0.534 g cm^-3^ |
| Number of Li anode layers (2 side coated on Cu foil) | 19 |
| Number of Li anode layers (1 side coated on Cu foil) | 2 |
| Density of LiCoO_2_ | 5.05 g cm^-3^ |
| Number of LCO cathode layers (2 side coated on Al foil) | 20 |
| Density of LLZO | 5.1 g cm^-3^ |
| Number of LLZO dense layers | 40 |
| Thickness of Al foil | 12 µm |
| Density of Al | 2.7 g cm^-3^ |
| Thickness of Cu foil | 12 µm |
| Density of Cu | 8.96 g cm^-3^ |
| Packaging foil | 50 µm |
| Areal weight of packaging foil | 10.5 mg cm^-2^ |
| Number of packaging foil layers | 2 |

**Table S3.** Comparison of the gravimetric and volumetric energy densities of the solid-state system (Li/LLZO/LLZO+LCO) at gravimetric and volumetric power density of 200 W kg^-1^ and 600 W L^-1^ (corresponding to *ca*. 1h of a full discharge) obtained using standard and expended simulations. The latter were performed by integrating additional parameters, such as LLZO/Li (0.1 Ω cm^2^) and LLZO/LCO (50 Ω cm^2^) interfacial resistances and the 13% porosity in LCO/LLZO solid-state cathode.

| **Standard simulations**  Cathode composition: 60 vol.% of LCO/40 vol.% of LLZO  T = 30°C; LLZO thickness = 30 µm;  **Expended simulations**  Cathode composition: 60 vol.% of LCO/27 vol.% of LLZO/13 vol.% of porosity  T = 30°C; LLZO thickness = 30 µm; | | | | |
| --- | --- | --- | --- | --- |
| Areal capacity, mAh cm^-2^ | Energy density, Wh kg^-1^  (standard simulation) | Energy density, Wh kg^-1^  (expended simulation) | ∆, Wh kg^-1^ | ∆, % |
| 0.5 | 62.2 | 62.2 | 0 | 0 |
| 1 | 105.5 | 108.9 | 3.4 | 3.22 |
| 2 | 158.3 | 165.9 | 7.6 | 4.8 |
| 3 | 187.2 | 197.5 | 10.3 | 5.50 |
| 4 | 203.7 | 214.0 | 10.3 | 5.06 |
| 5 | 211.2 | 216.0 | 4.8 | 2.27 |
|  | | | | |
| Areal capacity, mAh cm^-2^ | Energy density, Wh L^-1^  (standard simulation) | Energy density, Wh L^-1^  (expended simulation) | ∆, Wh L^-1^ | ∆, % |
| 0.5 | 329.0 | 323.7 | 5.3 | 1.61 |
| 1 | 550.4 | 542.0 | 8.4 | 1.53 |
| 2 | 813.8 | 799.5 | 14.3 | 1.76 |
| 3 | 959.9 | 939.4 | 20.5 | 2.14 |
| 4 | 1048.1 | 1017.2 | 30.9 | 2.95 |
| 5 | 1104.5 | 1054.2 | 50.3 | 4.55 |
